# A Systematic Review on the Influences of Neurotoxicological Xenobiotic Compounds on Inhibitory Control

**DOI:** 10.3389/fnbeh.2019.00139

**Published:** 2019-07-04

**Authors:** Cristian Perez-Fernandez, Pilar Flores, Fernando Sánchez-Santed

**Affiliations:** Department of Psychology and Health Research Center, University of Almería, Almería, Spain

**Keywords:** impulsivity, compulsivity, inhibitory control, lead, methylmercury, organophosphates, polychlorinated biphenyls

## Abstract

**Background:** Impulsive and compulsive traits represent a variety of maladaptive behaviors defined by the difficulties to stop an improper response and the control of a repeated behavioral pattern without sensitivity to changing contingencies, respectively. Otherwise, human beings are continuously exposed to plenty neurotoxicological agents which have been systematically linked to attentional, learning, and memory dysfunctions, both preclinical and clinical studies. Interestingly, the link between both impulsive and compulsive behaviors and the exposure to the most important xenobiotic compounds have been extensively developed; although the information has been rarely summarized. For this, the present systematic review schedule and analyze in depth the most important works relating different subtypes of the above-mentioned behaviors with 4 of the most important xenobiotic compounds: Lead (Pb), Methylmercury (MeHg), Polychlorinated biphenyls (PCB), and Organophosphates (OP) in both preclinical and clinical models.

**Methods:** Systematic search strategy on PubMed databases was developed, and the most important information was structured both in text and in separate tables based on rigorous methodological quality assessment.

**Results:** For Lead, Methylmercury, Polychlorinated biphenyls and organophosphates, a total of 44 (31 preclinical), 34 (21), 38 (23), and 30 (17) studies were accepted for systematic synthesis, respectively. All the compounds showed an important empirical support on their role in the modulation of impulsive and, in lesser degree, compulsive traits, stronger and more solid in animal models with inconclusive results in humans in some cases (i.e., MeHg). However, preclinical and clinical studies have systematically focused on different subtypes of the above-mentioned behaviors, as well as impulsive choice or habit conformations have been rarely studied.

**Discussion:** The strong empirical support in preclinical studies contrasts with the lack of connection between preclinical and clinical models, as well as the different methodologies used. Further research should be focused on dissipate these differences as well as deeply study impulsive choice, decision making, risk taking, and cognitive flexibility, both in experimental animals and humans.

## Introduction

### Rationale

Impulsivity is the inability to control a poorly predefined, risky and (often) inappropriate behavior in a specific context. At the same time, compulsivity is the persistence in an aimless, excessive and rigid action which could be considered maladaptive in changing contingencies (Moreno and Flores, 2012; Robbins et al., 2012; Fineberg et al., 2014). Both impulsivity and compulsivity are multidimensional constructs composed of a few distinguishable sub-domains within each endophenotype (Dalley et al., 2011; Robbins et al., 2012; Bevilacqua and Goldman, 2013; Fineberg et al., 2014; Dalley and Robbins, 2017).

Impulsive-related behaviors can be more accurately categorized based on functional cognitive tests on motor impulsivity (impulsive action), impulsive decision-making, impulsive choice and poor reflection rate. Compulsive-related sub-domains can be categorized into flexibility to contingency, attentional shifting, attentional bias/disengagement, and habit formation (Fineberg et al., 2014). In this way -albeit part of the same behavioral dimension- every single sub-domain can be individually studied and is affected in varying degrees in every single related disorder. Empirical data therefore supports both their inter-dependence and their independence (Fineberg et al., 2014).

The main neurocognitive tasks designed for impulsive choice assessment in preclinical research are the delayed discounting task, probabilistic, and temporal discounting tasks. Impulsive action is often evaluated with the 5-choice serial reaction time task (5C-SRTT, premature responding), differential reinforcement to low/high rates (DRL/H, prepotent responding), go no go task (GNGT, prepotent responding), stop signal task (SST, inability to stop initiated response), the simple reaction time task (SRTT) as well as different non-standardized operant schedules with fixed intervals (D'Amour-Horvat and Leyton, 2014).

Compulsivity is also assessed with 5C-SRTT (perseveration), scheduled-induced polydipsia (SIP, adjacent repetitive behavior), delayed alternation task (DAT, along with working memory component), marble burying task (MBT, along with anxiety), the trail making test (TMT, along with attention) as well as operant schedules by including both reversal and extinction phases (inflexibility to shifting contingencies) (Moritz et al., 2009; Thomas et al., 2009; Izquierdo and Jentsch, 2012; Angoa-Pérez et al., 2013; Navarro et al., 2017).

In clinical research, several both questionnaire-based and neurocognitive tasks are commonly used in order to assess these traits. Attending to impulsive choice and action analyzed with neurobehavioral tools, all the paradigms indicated for preclinical models are also used in humans, with the continuous performance test (CPT) as an alternative to the 5C-SRTT. Related functions such as risk taking are usually assessed with Iowa Gambling Task (IGT) (D'Amour-Horvat and Leyton, 2014).

Otherwise, compulsive perseveration and inflexibility is often assessed with shifting contingencies (reversal and extinction) with Wisconsin sorting card task (WCST) or DAT. The most important clinical scales and questionnaires for different impulsive and compulsive traits go from specific scales such as the Barratt impulsivity scale (BIS) or brief symptoms inventory (BSI) to general batteries such as the NEPSY or parent/teacher-referred indexes such as the Conners' rating scale or the Swanson, Nolan and Pelham scale (SNAP-IV) (Ahmad and Warriner, 2001; Reise et al., 2013; Crameri et al., 2016).

Different pathologies (psychiatric, neuropsychological, neurodevelopmental and neurodegenerative) have impulsive and/or compulsive traits as part of their central features, and they comprise the category of impulsivity/compulsivity spectrum disorders (Skodol and Oldham, 1996). Some of the most important related pathologies include obsessive-compulsive disorder (OCD), personality disorders, frontal lesions following vascular disease or traumatic brain injury, autism spectrum disorder (ASD), attention deficit/hyperactivity disorder (ADHD), idiopathic Parkinson's disorder, and Alzheimer's disease [Diagnostic and Statistical Manual of Mental Disorders (DSM-IV-TR); American Psychiatric Association, 2000].

Altered top-down regulation following mismatched fronto-striatal pathways, dysregulation of the basal ganglia and limbic regions, as well as the bottom-up influences of the monoaminergic system have been proposed as the physiological substrate of both impulsive and compulsive behaviors (Dalley et al., 2011; Fineberg et al., 2014; Dalley and Robbins, 2017).

On the one hand, the impulsive-related circuit has been linked to the anterior and inferior cingulate, ventromedial/lateral prefrontal cortex, medial/lateral orbitofrontal cortex, premotor cortex, hippocampus, infra/prelimbic cortices, ventral/dorsal striatum, and certain sub-thalamic areas (Dalley et al., 2011; Fineberg et al., 2014, 2018; Dalley and Robbins, 2017). Noradrenergic, serotonergic and dopaminergic systems are the most widely associated with impulsivity in an area/sub-domain-dependent manner, although gamma aminobutyric acid (GABA), glutamatergic and even endocannabinoid systems also play a significant role (Moreno et al., 2010, 2012; Dalley et al., 2011; D'Amour-Horvat and Leyton, 2014; Fineberg et al., 2014; Dalley and Robbins, 2017; Isherwood et al., 2017; Merchán et al., 2017; Dellu-Hagedorn et al., 2018).

On the other hand, compulsivity has been related to the dysregulation of both direct (ventral) and indirect (dorsal) cortico-striato-thalamo-cortical pathways (van den Heuvel et al., 2016; Fineberg et al., 2018), with the orbitofrontal cortex, the dorsal/ventral striatum as well as limbic regions such as hippocampus as essential structures (Moreno and Flores, 2012; Fineberg et al., 2014, 2018). Other areas such as the dorsolateral/lateral/ventromedial prefrontal cortex, the supplementary motor area, and the premotor cortex were also linked to these traits (Fineberg et al., 2014; Grant and Kim, 2014; Dalley and Robbins, 2017). Like for impulsivity, different neurotransmitter systems have been associated to compulsive behaviors. Cortico-striatal glutamatergic projection pathways in the regulation of GABA activity at dorsal striatum (van den Heuvel et al., 2016), 5-HT at the orbitofrontal cortex in relation to cognitive flexibility and dopamine in different frontal structures for flexibility and habit learning (Fineberg et al., 2018) have been proposed. The implication of the cholinergic system in compulsive polydipsia was also observed (Martín-González et al., 2018; Mora et al., 2018)

The involvement of genetics in impulsivity is set at around 20–60%, with some variability between subdomains and with interesting particularities related to age and gender, also depending on the assessment method (self-report scales vs. neurobehavioral measures) (Bezdjian et al., 2011; Bevilacqua and Goldman, 2013; Fineberg et al., 2014). Compulsivity heritability rates show little evidence of significant gene influence (Fineberg et al., 2014), although OCD epidemiological twin studies suggested an estimate of between 27 and 47% heritability in the adult population, with higher rates in children (≈ 55% variance) (Pauls, 2010), similar to other studies (Monzani et al., 2014).

Besides individual modulator of impulsive or compulsive behaviors, in the last decades also environmental modulators have been extensively studied. Specifically, researcher laid the focus to xenobiotic compounds, both natural and artificially produced. Lead (Pb) is a heavy metal widely used in multiple industrial and commercial products such as paints and cosmetics. It can be found in both organic and inorganic forms (Rana et al., 2018) and modulates different neurotransmitter systems such as GABA, dopamine, acetylcholine and glutamate with direct effects on N-methyl-D-aspartate (NMDA) ion channels as well as alters the regulation of intracellular calcium release (Mason et al., 2014; Chibowska et al., 2016).

In addition, methylmercury (MeHg) has an important industrial use and derived products (i.e., burning coal), although the most important sources of MeHg exposure for humans are fish and crustacean's consumption (Mergler et al., 2007; Li et al., 2014; Goodrich et al., 2016). Its neurotoxicological profile has been linked to different processes such as mitochondrial dysfunction, microtubule alterations, oxidative stress, intracellular calcium release, and concentration and lipid peroxidation (Karri et al., 2016; Zhang and Van Gestel, 2017). Some of these process apparently mediated by glutamatergic system dysfunction (Karri et al., 2016).

Furthermore, PCBs represent a heterogenous chemical group composed by more than 200 congeners (i.e., PCB 153, 126) and industrial mixes (i.e., Aroclor 1242, 1248, 1254 and 1260) characterized by strong persistence in the environment (Ribas-Fito et al., 2001). The main sources of exposure in humans go from fish and animal fats consumption to industrial and commercial products. The neurotoxicological effects of this family of xenobiotics highly depend on the planarity of the specific congener attending to the capacity to bind the aryl hydrocarbon receptor (planar or coplanar vs. non-planar/coplanar PCBs). This depends on the disposition of the chlorine atoms at the molecule. Interestingly, these congeners with low affinity to aryl hydrocarbon receptor also have a toxicological profile by interacting with other proteins (Fischer et al., 1998). All these compounds can induce negative effects on health by multitude molecular mechanisms such as intracellular activity mismatching (second messengers, calcium dependent processes, kinases activity) as well as different neurotransmitter systems such as dopamine, serotonin, GABA, amongst others (Choksi et al., 1997; Tilson and Kodavanti, 1998; Boix and Cauli, 2012).

Finally, OP compounds are a wide range of pesticides commonly used for agricultural, public/residential and industrial purposes. Their main mechanism of action is the irreversible inhibition of different classes of cholinesterase (ChE), particularly acetylcholinesterase (AChE) at the CNS (Fukuto, 1990). However, alternative targets such as direct impacts on other neurotransmitter systems than cholinergic system (5HT, Dopamine and Endocannabinoid systems, amongst others), other proteins into the cholinergic system (such as nicotinic and muscarinic receptors, amongst others), mitochondrial function alterations, oxidative stress, and lipid peroxidation have been proposed (Akbel et al., 2018). From all the OP compounds found in the current market, Chlorpyrifos (CPF) is the most widely used the last decades (Eaton et al., 2008).

The well-documented negative effects on health and cognitive functioning following exposure to these agents is gaining interest in the last decade due to their widespread use and environmental persistence (Safe, 1994; Burns et al., 2013; Sánchez-Santed et al., 2016; Abreu-Villaça and Levin, 2017). Added to this, all these compounds have a stronger toxicological profile when exposure occurs during the development stage, where several research groups have been focusing their interest on in the last decades (i.e., Ribas-Fito et al., 2001; Hu et al., 2006; Myers et al., 2015; Hertz-Picciotto et al., 2018). However, and to the best of our knowledge, there are no systematic reviews specifically focusing on the relations between these hazardous compounds and impulsive and compulsive outcomes, neither human nor preclinical models. Only few systematic and classical reviews partially touched this issue, mostly focused on impulsive traits and subtypes in ADHD, ASD patients, and suicide behaviors (London et al., 2005; de Cock et al., 2012; Freire and Koifman, 2013; Polanska et al., 2013; González-Alzaga et al., 2014; Daneshparvar et al., 2016).

### Objectives

The main objective of the present review was to systematically analyze, schedule, and critically study the different works focused (both as a central or secondary role) on some of the 4 xenobiotic compounds described above in relation to the different impulsive and/or compulsive subtypes in both preclinical and clinical fields. The four agents were selected as they are the most widely used hazardous xenobiotic compounds of the last decades and come from different sources of exposure in the environment.

### Research Question

Attending to the rationale and the main objectives proposed, our main questions are based on 2 different aspects: (1) Does the exposure to the different xenobiotic compounds actually increase impulsive and/or compulsive behaviors in both human and animal models? and (2) Are both preclinical and clinical fields working in the same direction with a translational perspective or are they completely unrelated?

## Selection Procedures and Search Strategy

### Study Design

The present manuscript represents a systematic review of the most important empirical works published in English in relation to the exposure to different hazardous agents and their effects on impulsive and/or compulsive outcomes. Two different selection checklists (one for preclinical and another for human studies) were design following general Cochrane's guidelines for studies acceptance. After initial evaluation, once a study successfully fulfilled essential criteria to get included into the present review, a deep quality assessment protocol was undergone. Both final selection and quality assessment was done by two of the authors individually. All discrepancies were solved in a single meeting by analyzing the affected studies and discussing the different points of view.

### Participants, Interventions, Comparators

The participants of the different experiments were: Humans (children, adolescents and adults), monkeys (Macaca fascicularis, Rhesus monkeys and Squirrel monkeys), rats (Sprague Dawley, Wistar, Long Evans, amongst others), and Mice. Interventions were varied and are systematically described into the different tables (Supplementary Tables 1–4).

Comparators in preclinical studies were animals exposed to different vehicles in the same fashion that the experimental ones. In the case of the clinical studies, most of the studies did not include acceptable comparators (i.e., longitudinal studies which correlated xenobiotic concentration with neurobehavioral performance), although some works used control groups with non to little exposed participants (non-randomized controlled studies).

### Systematic Review Protocol, Search Strategy, Data Sources, Study Selection

The systematic review was as follows: It was exclusively done by using PubMed as database. No date limit was set in order to select a study. For animal studies, we limited the search to murines and non-primate humans. Reviews, meta-reviews, systematic reviews, letters to editor and single cases were discarded. Only works in English were chosen.

The following words**:** *Pb, Methylmercury, MeHg, Polychlorinated Biphenyls, PCBs, Organophosphates, Chlorpyrifos* were individually mixed with the following functions and tasks: *Inhibitory control, impulsivity, impulsive choice, impulsive action, motor impulsivity, decision-making, risk-taking, premature, discounting, Go/NoGo, stop signal, continuous performance, compulsivity, compulsive, flexibility, inflexibility, perseverative, perseveration, extinction, 5C-SRTT, reversal learning, marble burying, alternation, stroop* and *trail making*. We noticed that early works used operant schedules with no standard nomenclature; we added the concept “*operant*.” The concept “*lead*” was not used due to the extremely high result rate, as expected.

The selection protocol was based on the reading of the abstracts and titles. When the abstract included some behavioral outcome in relation to our compounds, we included the study in the next phase. After this, duplicated works were discarded. All the studies were then deeply analyzed with respect to their methodological quality. The study had to have one impulsive and/or compulsive sub-trait as an essential or secondary scope of the paper, always in relation with one or more of the compounds studied.

In some cases, such behaviors included here as impulsive or compulsive outcomes were not explicitly considered liked that by the authors, but a more general inhibitory control performance or even mixed with learning or attentional functioning. Those studies which fulfilled a minimum of methodological quality were introduced into the final analyses. Their main characteristics are systematically described into the different tables. Only a few studies were recruited parallel to the main PubMed searching, based on their relevance in relation to our scope. They were acquired from other referenced papers. The last day of searching was the 14th January 2019. Finally, the quality of all the studies was deeply analyzed.

### Systematic Study of the Internal Validity (Quality) of Each Selected Study

The systematic analysis of the quality of all the included studies was conducted based on two models which generated a global quality index: (1) the method's section analysis as described in the Strengthening the Reporting Studies in Epidemiology (STROBE) statement checklist (von Elm et al., 2008), and (2) the specific analysis of the six main bias sources as referred by the Cochrane's guidelines.

Briefly, STROBE statement checklist is composed by a total of 22 (9 in methods section) individual and independent subsections (items), previously used in other systematic reviews focused on neurotoxicological effects of different xenobiotic compounds on behavior (i.e., González-Alzaga et al., 2014). Although it was designed for observational studies, the adaptation for experimental studies such as the preclinical works analyzed here was basically needed for the STROBE's subsection referred to participants and study design (Item 6).

However, and as pointed out in the Cochrane's guidelines, bias control is the most important factor in order to ensure proper quality in both randomized and non-randomized studies. Although STROBE statement checklist also gives importance to this, bias control score is only 1 out of the 9 items at the method's analysis. In order to solve this, we eliminated this and made a deep analysis of each of the six bias sources from Cochrane: Selection bias (random sequence generation and allocation concealment), performance bias (blinding of participants and personnel), detection bias (blinding of outcomes assessment), attrition bias (incomplete outcome data), and reporting bias (selective reporting). This gave us a score range from 0 to 6 (one point per bias control). The STROBE gave us a score range from 0 to 8 (from the 8 remaining items of methods section after removing bias subsection).

For total quality score, we simply added these two rates, generating a range from 0 (null quality) to 14 (very high quality). That is to say, the initial relative contribution of bias control to the STROBE (1/9, 11%) turned to almost 4 times more relevance following this assessment protocol (6/14, 43%). For the total quality score, ranges were from 0 to 3, 4 to 5, 6 to 7, 8 to 9, 10 to 12, 13 to 14 for low (L), medium-low (ML), medium (M), medium-high (MH), high (H) and very high quality, respectively. Added to this, sample size of each study was also included, with a “+” symbol when the sample size was appropriate (≥8), “–” when inappropriate and “?” when the number of animals/group was not explicit, or we were not able to find this information.

## Results

### Flow Diagram of Systematic Searching Protocol and Studies Selection

**Figure 1:**
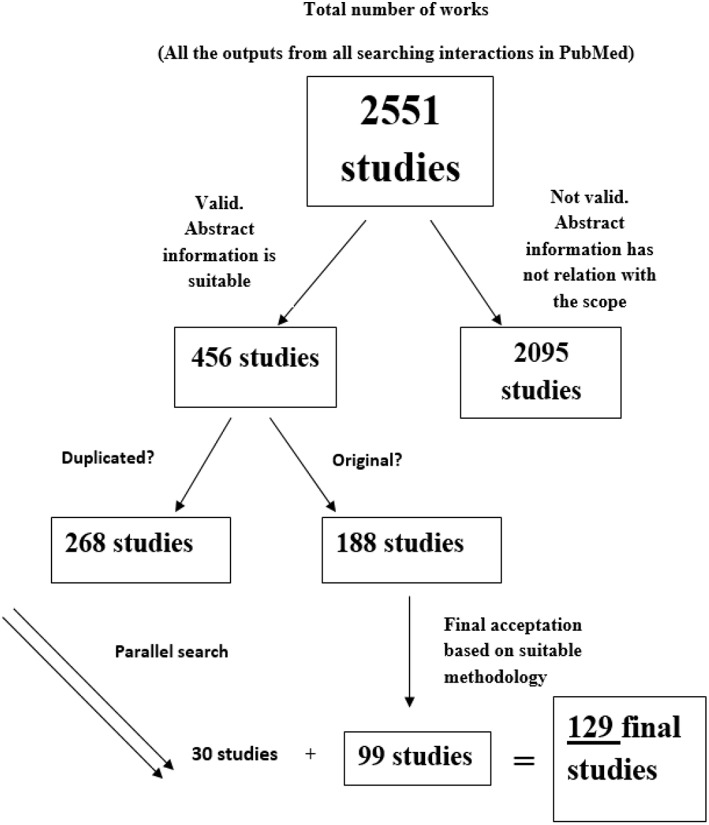
Diagram flow. From top to bottom, total number of outputs from PubMed searching (*n* = 2,551), number of studies selected based on apparent suitable abstract (*n* = 456), total number of apparent suitable abstract when duplicated works were excluded (*n* = 188), total number of studies which successfully passes the selection checklist (*n* = 99) and final total number of accepted studies after parallel searching (*n* = 129).

The flow diagram following the whole output from the beginning to the last selection is displayed in the Figure 1. Our first screening of total results drove to 2,551 studies. Once we read the abstracts, sample was to 456 possible candidates, letting 188 original studies after duplicates were discarded. Following this, a deep analysis focused on the study of the description of the behavioral task described on each paper reduced the sample to 99 papers. However, we found more studies based on parallel searching by analyzing the references of the above-mentioned works. The final number of analyzed studies was 129.

### Internal Validity and General Quality

Sample size in preclinical studies was generally acceptable except for most of the studies with monkeys (i.e., Bushnell and Bowman, 1979a,b; Rice et al., 1979; Cory-Slechta et al., 1981; Levin et al., 1987; Levin and Bowman, 1988; Newland et al., 1994; amongst others). To this, around 15% of the studies used low to very low animals per group. All clinical studies used a proper sample size, in some cases with very large sample (i.e., Zhang et al., 2009; Sagiv et al., 2010, 2012; Hong et al., 2015; Joo and Roh, 2016).

Although bias checking was the same for both clinical and preclinical studies, slight differences in the score criteria of some items in the STROBE invalidate the proper comparisons between fields (i.e., a high-quality clinical study does not mean that it is better done than a medium-high quality preclinical work). On this way, we were severer in the penalization of the lack of explicit randomization in preclinical studies, something which affected to the item related to participants and study design (item 6) and, in some circumstances, the item associated with the proper explanation of how study size was arrived at (item 10). Furthermore, the control of confounding variables (item 7) was common and properly done in most of the clinical studies (the control of the main confounding variables such as age, education level, etc.). However, we set motor and/or motivational outcomes as the essential control assessment in preclinical works, something rarely implemented in most of the studies.

For preclinical studies (89), the lowest valuated work was Rice et al. (1979), with 5 points out of 14, meanwhile 3 studies were classified as high quality with a general valuation of 12 out of 14 (Lilienthal et al., 1990; Garavan et al., 2000; Maurissen et al., 2000). We found neither very high nor low works. The 18% of the studies were categorized as medium-low, meanwhile the 27% and the 32% were labeled as medium and medium-high, respectively. Finally, the remaining studies (24%) were classified as high quality. Otherwise, the worst valuated paper across clinical studies (39) was Sánchez Lizardi et al. (2008). On the other hand, two studies obtained the highest rate with 11 out of 14 (Marks et al., 2010; Wesseling et al., 2010). Only one study showed low-quality as mentioned above, meanwhile the 10%, the 13% and the 59% were classified as medium-low, medium and medium-high quality, respectively. The remaining 15% were labeled as high-quality.

### Main Findings

#### Natural Xenobiotic Compounds (The Case of Two Ubiquitous Environmental Contaminants)

##### Lead

A total of 31 preclinical studies were included in relation to both impulsivity and compulsivity traits (Supplementary Table 1a). From these, 4 (13%) were classified as high, 5 (16%) as medium-high, 12 (39%) as medium and 10 (32%) as medium-low quality. Attending to sample size, 15 (48%) used low to very low animals per group. Otherwise, at clinical level, a total of 13 studies were accepted for review, with 2 (15%) labeled as high, 9 (69%) as medium-high, 1 (8%) as medium and 1 (8%) as medium-low quality (Supplementary Table 1b). All of them with a proper sample size.

##### Impulsivity and Lead

Only one out of 11 works (9%) was categorized as medium-high (Cory-Slechta et al., 1981), 6 (55%) as medium (Cory-Slechta and Thompson, 1979; Rice and Gilbert, 1985; Cory-Slechta et al., 1996, 1998, 2002; Brockel and Cory-Slechta, 1998; Stangle et al., 2007), and 4 (36%) as medium-low quality (Rice et al., 1979; Angell and Weiss, 1982; Cory-Slechta et al., 1985; Rice, 1992).

Despite some early research did not find increased responding rates following Pb exposure (Rice and Gilbert, 1985), other such as Rice (1992) found increased impulsive action in adult monkeys exposed from PND1 by inefficient responding pattern in a DRL task, particularly at later stages. Similarly, Cory-Slechta et al. (1981) observed that rats chronically exposed to Pb from weaning increased the total number of responses, shortening their duration in a modified DRL-like paradigm and showing insensitivity to external auditory facilitation.

Cory-Slechta and Thompson (1979) early found increased responding rate on a fixed interval schedule of reinforcement in rats exposed to 50–300 ppm/day from weaning. This was also observed following 25 ppm by shortening inter response times in a later study (Cory-Slechta et al., 1985). However, effects after weaning have not been systematically observed in rats (Angell and Weiss, 1982). Otherwise, early post-natal exposure increased response-rate in rats, which can be added to the increased response-rate and response-burst pattern observed in Rice et al. (1979) in monkeys exposed from PND1.

These early post-natal exposure effects were also observed in other fixed interval reinforcement paradigms, showing greater rates of responding per minute (Cory-Slechta et al., 1998) and lower post-reinforcement pauses (Cory-Slechta et al., 2002) in exposed male rats. Moreover, the literature supports that the dopamine system, particularly in the ventral and dorsomedial striatum, is implicated in the fixed interval altered behavior following Pb exposure. These findings are like the increased impulsivity and inefficient behavioral response patterns (Multiple fixed ratio waiting-forward schedule of reinforcement) observed by Brockel and Cory-Slechta (1998) in rats. This dopamine implication on the regulation of impulsive traits on Pb exposure is not surprising (Cory-Slechta et al., 1996).

Attending to the studies carried out with humans (10), 2 of them (20%) were labeled as high (Ethier et al., 2015 and Nigg et al., 2010), 7 (70%) as medium-high (Stewart et al., 2003, 2006; Nicolescu et al., 2010; Plusquellec et al., 2010; Boucher et al., 2012a,b; Hong et al., 2015) and 1 as medium quality (Lee et al., 2018).

Although Ethier et al. (2015) found that gestational but not concurrent exposure (exposure at the time of testing) to Pb was linked to greater commission error rates (visuospatial attention-shift paradigm), other studies found that higher Pb levels in both gestational and post-natal measures also increase impulsive action rates in a GNGT (Boucher et al., 2012a). This behavioral alteration following both developmental stages was also found in a CPT (Stewart et al., 2003). In contrast, Boucher et al. (2012b) found that postnatal but not gestational Pb exposure levels were associated with hyperactivity/impulsivity type ADHD symptomatology, supported by both previous and recent studies (Nigg et al., 2010; Lee et al., 2018).

Added to this, Hong et al. (2015) found a positive correlation between postnatal Pb exposure levels and impulsivity rates (both questionnaire and neurobehavioral-based) in children. These postnatal-linked findings were also supported by the results from Nicolescu et al. (2010) in pre-adolescent children and Stewart et al. (2006) in children. Finally, low levels of postnatal (but not gestational) exposure positively correlated with greater parent-reported impulsive behavior in Inuit children (Plusquellec et al., 2010).

##### Compulsivity and Lead

In animal studies, 21 works analyzed the relation of Pb exposure with flexibility and perseveration outcomes. 4 of them (19%) were labeled as high (Hastings et al., 1984; Cohn and Cory-Slechta, 1993, 1994; Garavan et al., 2000), 4 (19%) as medium-high (Bushnell and Bowman, 1979a; Rice and Gilbert, 1990a; Hilson and Strupp, 1997; Lim et al., 2005), 6 (29%) as medium (Levin and Bowman, 1986, 1988; Rice and Karpinski, 1988; Newland et al., 1994; Cory-Slechta et al., 2002; Chang et al., 2014) and 7 (33%) as medium-low (Bushnell and Bowman, 1979b; Rice et al., 1979; Rice, 1985, 1990, 1992; Gilbert and Rice, 1987; Rice and Gilbert, 1990b).

Gestational Pb exposure in monkeys was linked to insensitivity to shifting contingencies by perseverative responding even 5–6 years after exposure (Newland et al., 1994). Earlier works such as those of Bushnell and Bowman (1979a) as well as Rice et al. (1979) also found increased inflexibility (increased responding at time out periods) and impaired learning after Pb exposure in different monkeys in a serie of discriminative reversal learning tasks, effects with long-term persistence (Bushnell and Bowman, 1979b). Rice (1985) also found greater inflexibility rates on non-spatial discrimination tasks (color, form, and with the influence of irrelevant cues) in chronically exposed from PND1 monkeys. In addition, Gilbert and Rice (1987) as well as Rice and Gilbert (1990a) observed that the introduction of irrelevant cues increased such impairments, also with primates.

Moreover, concurrent exposure appeared to be more critical than early exposure in inflexibility-related behaviors, although early exposure exacerbated such alterations. Following this, Rice (1990) found similar alterations in spatial discrimination paradigms in monkeys, with both relevant and irrelevant cues. Interestingly, although all groups were impaired following the introduction of novel irrelevant cues, only animals exposed from birth to onwards were affected on the normal task. However, some examples of preserved flexibility following Pb exposure were found, specifically in rats (i.e., Hastings et al., 1984).

Similar perseveration/lack of inhibition/effects of irrelevant cues were found following a DAT in monkeys exposed to Pb from PND1 (Rice and Karpinski, 1988). However, these task-related alterations were insensitive to age of exposure (Rice and Gilbert, 1990b). These results agree with those previously reported by Levin and colleagues, where chronically exposed monkeys but with scheduled high pulses of Pb for the first year of life systematically performed the DAT with higher rates of perseveration (Levin and Bowman, 1986). Interestingly, this was not found when chronic exposure did not consist of high pulse dosage stages (Levin and Bowman, 1988). In addition, other studies failed to find such alterations in DAT in monkeys when using larger dosages (Rice, 1992).

Once again, dopamine system seems to play an essential role in the regulation of these behaviors (Levin et al., 1987), as well as in the regulation of other compulsive traits in rats (Chang et al., 2014). Furthermore, the glutamatergic system was linked to specific modulations of perseverative responding in exposed rats from weaning (Cohn and Cory-Slechta, 1993, 1994). However, other authors proposed that these altered patterns resulting from the reversal task could be not due to impaired inhibition or inflexibility because abnormal behavior in exposed animals was found at late stages after reversal. After controlling the different post-reversal stages, they concluded that other cognitive functions (attentional, learning, or associative) rather than inflexibility could be at the basis of Pb-related impairments (Rice, 1985; Hilson and Strupp, 1997; Garavan et al., 2000).

Attending to the clinical field, we found only 4 acceptable studies for this scope, 3 of them (75%) labeled as medium-high (Stiles and Bellinger, 1992; Surkan et al., 2007; Nicolescu et al., 2010) and the remaining (25%) as medium-low quality (Evans et al., 1994).

Stiles and Bellinger (1992) found that both early (2 years old) and late (10 years old) postnatal Pb levels positively correlated with greater perseverative outcomes in children at the age of 10 assessed with the WCST and the California Verbal Learning Test for Children. Similarly, Evans et al. (1994) found that postnatal exposure levels were associated with both learning and flexibility impairments in children (twins), as indicated by a greater number of errors during acquisition of a visual discrimination task and a reversal condition. Furthermore, an increased number of perseverative responses (WCST) was associated with lower level of Pb exposure (5–10 ug/dL) in children (Surkan et al., 2007). Finally, Nicolescu et al. (2010) also found increased compulsive responses, as shown by altered flexibility rates in exposed children.

##### Methyl-Mercury (MeHg)

The systematic analysis of the studies of this section is shown in Supplementary Tables 2a,b. A total of 21 preclinical studies were included for analysis. From these, 5 (24%) were categorized as high, 10 (48%) as medium-high and 6 (28%) as medium quality. 7 (33%) used low to very low sample size. On the other hand, a total of 13 clinical studies were included for the final analysis, 3 (23%) high, 8 (62%) medium-high and 2 (15%) medium quality, with proper sample size in all cases.

##### Impulsivity and MeHg

Nine out of those 21 works specifically studied impulsive outcomes. From these, 2 studies (22%) were labeled as high (Paletz et al., 2006; Boomhower and Newland, 2016), 5 (56%) as medium-high (Rasmussen and Newland, 2001; Reed and Newland, 2007, 2009; Reed et al., 2008; Sable et al., 2009) and 2 (22%) as medium quality.

Increased impulsive choice (delayed discounting task) in adulthood was linked to postnatal MeHg exposure during the entire adolescent period in mice (Boomhower and Newland, 2016). In terms of motor impulsivity, as Paletz et al. (2006) observed that gestational/perinatal MeHg exposure increased responding rate as well as triggered an inefficient behavioral pattern in rats at early stages of a DRL task. This impulsive action pattern was also observed under progressive rate escalation at low response demands requirement. Moreover, Reed et al. (2008) found sensitization to reinforce magnitude in exposed rats.

Furthermore, Reed and Newland (2007) also introduced an external stimulus in order to generate a “clock” control of fixed interval operant behavior, and still found that the high exposure group showed generally high rates of impulsivity on both clocked and un-clocked components. This exposure protocol also triggered long-term DA system sensitivity, effects that were found independently of DA 1/2 receptor regulation. The importance of the DA system in the regulation of impulsive/compulsive traits following MeHg exposure has strong empirical support (Newland et al., 2008). For instance, Reed and Newland (2009) found altered DA system in differential reinforcement to both clocked and un-clocked components increased sensitivity to cocaine administration following gestational/perinatal MeHg exposure.

Otherwise, a hypo-sensitive GABAergic system in MeHg exposed rats was also found after pentobarbital challenge as well as an increase in impulsive action by aging (Newland and Rasmussen, 2000; Rasmussen and Newland, 2001). Similar alteration of the DA system was also found in other works (Sable et al., 2009). Whilst the latter authors did not find altered DRL performance in rats exposed to MeHg (both gestational and postnatal), the co-exposure of MeHg with amphetamine was less disruptive in MeHg groups in comparison with control rats.

Attending to human studies, all works except 1 (12) did study impulsivity-linked variables in relation to MeHg exposure. From these, 2 (17%) were classified as high (Ethier et al., 2015; van Wijngaarden et al., 2017), 8 (66%) as medium-high (Stewart et al., 2003, 2005, 2006; Yokoo et al., 2003; Nicolescu et al., 2010; Plusquellec et al., 2010; Boucher et al., 2012a,b) and 2 (17%) as medium quality (Debes et al., 2006; Lee et al., 2018).

All these preclinical findings on impulsive action have also been observed in children using a DRL task following gestational exposure to MeHg by earning less money whilst producing faster responses (Stewart et al., 2006). However, other studies found that gestational exposure could be more linked with attentional deficits (based on teacher reports) than hyperactive/impulsive traits (Boucher et al., 2012b). In an earlier study, Yokoo et al. (2003) also found a positive correlation between levels of exposure to MeHg and commission errors in an attentional task in adult population.

In a recent study, Lee et al. (2018) explored the effects of postnatal exposure on different types of ADHD (attentional and hyperactive/impulsive) in children and observed significant positive correlation between Hg levels and parental reports of hyperactive/impulsive traits. Interestingly, a recent meta-analysis conducted by Yoshimasu et al. (2014) summarized the relation between Hg (organic and inorganic forms) with both ADHD and ASD. Authors found moderate negative correlations between environmental exposure to such agents and both developmental pathologies.

However, the relation between MeHg gestational exposure levels and increased impulsive action has not been systematically found in humans (Stewart et al., 2003, 2005), as well as for postnatal (Nicolescu et al., 2010) or both gestational and postnatal (Debes et al., 2006; Plusquellec et al., 2010; Ethier et al., 2015; van Wijngaarden et al., 2017).

##### Compulsivity and MeHg

At preclinical level, 14 out of 21 studies did include compulsive outcomes in relation to MeHg exposure. From these, 3 (21%) were high (Doré et al., 2001; Goulet et al., 2003; Boomhower and Newland, 2017), 6 (43%) medium-high (Goldey et al., 1994; Newland et al., 2004; Widholm et al., 2004; Weiss et al., 2005; Onishchenko et al., 2007; Reed and Newland, 2007) and 5 (36%) were medium quality (Gilbert et al., 1993; Newland et al., 1994, 2013; Reed et al., 2006; Paletz et al., 2007).

An early study by Newland et al. (1994) found long-term insensitivity to changing contingencies in monkeys following gestational MeHg exposure. Reed et al. (2006) found that gestational/perinatal exposure to MeHg increased perseverative responding on a spatial discrimination task in rats at early reversal phases. Like this, inflexibility was also observed in exposed mice following reversal stages in place-learning paradigms (Onishchenko et al., 2007). Furthermore, Paletz et al. (2007) also found these alterations in rats in both spatial and visual discrimination schedules at early reversal stages and in a dose-dependent manner.

Interestingly, other studies have found that the transition from DRH to DRL increased inflexibility/perseveration in MeHg exposed (gestational/perinatal) in a dose-dependent manner (Newland et al., 2013). Similarly, Reed and Newland (2007) also found increased perseveration (high response rates at reinforce omission stage) in both clocked and un-clocked fixed interval components in high exposed rats. Interestingly, aging appears to be an important factor in MeHg effects on cognitive function and, specially, in perseveration (Newland et al., 1994, 2004; Paletz et al., 2007).

Performance of exposed rats on delayed/alternation task has also been explored. On this way, MeHg exposure for short periods during gestation was linked with a significant decrease in DAT performance without affecting motor activity in mice (Doré et al., 2001). Moreover, other studies found that both perinatal and concurrent exposure have an impact on DAT performance, leading exposed animals to appropriately unlearn the task, particularly at longer delays (Weiss et al., 2005). DAT alterations were also observed in rats exposed during gestational and perinatal stages, along with perseveration in a spatial reversal learning task (Widholm et al., 2004).

However, an early study conducted by Gilbert et al. (1993) found that adult monkeys exposed to this agent throughout pregnancy performed even better than their control counterparts. A similar lack of effects was found following both gestational (Goldey et al., 1994) and gestational-postnatal exposures in rats (Goulet et al., 2003). Finally, Boomhower and Newland (2017) recently found increased “perseverative” errors at the second reversal, thus attentional/associative influences could fit better than inflexibility in this case.

Unfortunately, the effects MeHg on compulsive behavior have rarely been studied, with 3 main works with both high (Philibert et al., 2008; van Wijngaarden et al., 2017) and medium-high quality labeling (Nicolescu et al., 2010). Both Philibert and van Wijngaardeen's studies found significant negative influences of postnatal MeHg exposure in compulsive traits (the obsessive/compulsive sub scale of the BSI in adult women and errors from intra/extra dimensional shift in children, respectively). Otherwise, Nicolescu's study did find no association between inflexibility rates and postnatal Hg levels, like in the case of impulsive outcomes above mentioned.

#### Artificial Xenobiotic Compounds

##### PCBs

The systematic analysis of the studies in this section is shown in Supplementary Tables 3a,b. A total of 23 preclinical studies were included, 8 of them (35%) classified as high, 8 (35%) as medium-high, 2 (8%) as medium and 5 (22%) as medium-low quality. Two of them used low to very low sample size per group. Otherwise, a total of 15 clinical studies were included, categorized as high (1, 7%), medium-high (13, 86%), medium quality (1, 7%), all of them with proper sample size.

##### Impulsivity and PCBs

With regard to animal studies, a total of 15 works studied impulsive, 5 (33%) labeled as high (Lilienthal et al., 1990; Holene et al., 1998; Rice and Hayward, 1998; Berger et al., 2001; Lombardo et al., 2015; Monaikul et al., 2017), 6 (40%) as medium-high (Rice and Hayward, 1999b; Sable et al., 2006, 2009; Johansen et al., 2011; Meyer et al., 2015), 2 (13%) as medium (Rice, 1997, 1998) and 2 (13%) as medium-low (Rice and Hayward, 1999a; Johansen et al., 2014).

Lilienthal et al. (1990) found increased response rates between reinforcements (continuous reinforcement schedule test) in rats exposed to high dietary doses of PCBs, both gestational and postnatal. Following this, Rice (1997) found reduced inter response time and pauses on a multiple fixed interval schedule of reinforcement in postnatal exposed monkeys. Similarly, Rice (1998) also observed an altered learning pattern on a DRL task in postnatal exposed monkeys, with lower inter response time and reinforces gained, as well as hyperactivity.

In addition, both gestational and postnatal exposure to PCB increased reinforced responses, decreased response duration and shorter inter response time during DRH schedule in rats (Sable et al., 2006). Sable et al. (2009) found increased motor impulsivity (ratio of reinforced/non-reinforced responses) following PCB developmental exposure in DRL with larger effects for males. Added to this, a recent study conducted by this same group also found increased impulsive action and shorter inter response time in PCB exposed rats using the same exposure protocol, but with female rats (Meyer et al., 2015).

Otherwise, Holene et al. (1998) found that male rats postnatal exposed to both PCB-153 and 126 congeners were more impulsive and hyperactive as well as less efficient in reward collecting, with greater burst responding for PCB-153 exposed animals. Increased motor impulsivity and hyperactivity following this congener was also found in female hypertensive rats (Johansen et al., 2014). However, PCB-52 congener but not 153 or 180 decreased performance (variable schedule of reinforcement) in postnatal exposed adolescent rats (Johansen et al., 2011). In fact, this hyperactive and impulsive action pattern observed was also found following the mix Aroclor 1248 exposure during adolescence/early adulthood in adult male rats (Berger et al., 2001; Lombardo et al., 2015).

Finally, PCB negative effects on impulsive traits have not been always proved. On this way, postnatal PCB exposure had little effect on a progressive fixed ratio schedule (Rice and Hayward, 1999a). Related to this, Rice and Hayward (1998, 1999b) did not find significant alterations in both fixed interval/ratio and DRL or both multiple random intervals and progressive ratio reinforcement schedules following both gestational and early postnatal exposure to PCB-126 in rats. This lack of influence on DRL was supported by Sable et al. (2006), which involved perinatal PCB exposure, and other more recent works such as Monaikul et al. (2017), which involved adolescent PCB exposure.

In human studies, a total of 14 studies analyzed impulsive outcomes in relation to different PCB congeners, 1 (7%) of them was labeled as high (Ethier et al., 2015) and the remaining 13 (93%) as medium-high (Jacobson and Jacobson, 2003; Stewart et al., 2003, 2005, 2006; Vreugdenhil et al., 2004; Plusquellec et al., 2010; Sagiv et al., 2010, 2012; Boucher et al., 2012a,b; Verner et al., 2015; Behforooz et al., 2017).

Stewart et al. (2003) found that higher rates of gestational exposure to PCBs were related to impaired response inhibition (adapted vigilance task) in children, linked with a significant reduction in the splenius of the corpus callosum volume. These authors also observed similar effects at older ages in the same cohort of children (Stewart et al., 2005, 2006). Both gestational and postnatal PCB levels were also linked to clinical symptomatology of ADHD, specifically impulsivity and hyperactivity outcomes, albeit gestational exposure played a more solid role (Verner et al., 2015).

Furthermore, Jacobson and Jacobson (2003) found that gestational exposure to PCB influences on impulsivity was modulated by breastfeeding time. Authors observed that exposed pre-adolescent children made more commission errors in a CPT (thus showing general impulsivity), but only when they had received <6 weeks of breast milk feeding. This protective effect of breastfeeding against PCB related alterations has also recently been observed in a Spanish cohort (Forns et al., 2012), although the authors did not find significant relationships between PCB levels and impulsive traits (CPT).

Finally, Sagiv and colleagues also linked gestational PCB exposure with ADHD-like symptomatology in children, although this seems to be more strongly associated with attentional traits than the inhibitory control dimension, particularly in boys (Sagiv et al., 2010, 2012). Similar results were found in a recent study conducted by Behforooz et al. (2017). The results of this study revealed a positive correlation between PCB exposure rates and altered attention in young male adults, with no significant effects regarding impulsive domains (Conner's CPT-II), like other previous studies (Ethier et al., 2015). This lack of association between PCB exposure levels and impulsive behavior has also been found elsewhere (Plusquellec et al., 2010; Boucher et al., 2012a,b).

##### Compulsivity and PCBs

A total of 13 preclinical studies analyzed compulsive outcomes. From these, 4 (31%) were classified as high (Holene et al., 1998; Rice, 1999; Zahalka et al., 2001; Monaikul et al., 2017), 5 (38%) as medium-high (Schantz et al., 1996, 1995, 1989; Berger et al., 2001; Sable et al., 2006), 1 (8%) as medium (Rice, 1998), and 3 (23%) as medium-low quality (Bowman et al., 1978; Rice and Hayward, 1997; Widholm et al., 2001).

Early preclinical studies found that monkeys following gestational/early postnatal exposure to Aroclor 1248 increased perseverative and inflexible behavior patterns on spatial, color reversal tasks and shifting stages in probabilistic tasks, but also general hyperactivation (Bowman et al., 1978). Rice and Hayward (1997) found increased long-term inflexibility (non-spatial discrimination reversal tasks) and altered DAT acquisition in monkeys given postnatal PCBs. More recently, high rates of perseveration during the extinction (DRL task) were also observed in high PCB exposed rats during development (Sable et al., 2006).

Interestingly, Holene et al. (1998) also found this during extinction phases of a fixed interval schedule of reinforcement but following into the first two postnatal weeks. In addition, continuous gestational/postnatal exposure until weaning to Aroclor 1254 increased perseverative responses in exposed male rats at the first reversal series, whilst exposed females showed this at later stages (Widholm et al., 2001). In this case, responding in females could be strongly linked to attention/learning deficits than compulsivity.

Contrary, exposure to Aroclor 1248 mix enhanced performance on spatial reversal tasks with shape as the irrelevant stimuli (Schantz et al., 1996). Like this, Aroclor 1248 mix did not induce specific alterations on extinction phase following fixed interval schedule of reinforce, even though it generally increased impulsive rates (Berger et al., 2001). Following this, 1016 compound also impaired learning of spatial tasks without affecting reversal (Schantz et al., 1996), like later research in spatial tasks (Rice and Hayward, 1998) and intermittent schedules (Rice and Hayward, 1999b). Moreover, some studies found improved perseverative rates following PCB exposure (Monaikul et al., 2017). Finally, lack of effects on perseveration performance (DAT) was also observed in rats following perinatal (gestational and postnatal) exposure to PCB-126 congener and others (Rice, 1999; Zahalka et al., 2001).

Once again, human studies are less prominent. On this way, only 2 studies were found in relation to compulsive traits, labeled as medium-high (Jacobson and Jacobson, 2003) and medium quality (Fimm et al., 2017). Following impulsive patterns previously described, Jacobson and Jacobson (2003) found that gestational exposure to PCBs in relation to breastfeeding increased compulsive responses, as assessed by WCST in children. However, Fimm et al. (2017) did not find significant altered flexibility or attentional domains in relation to PCB exposure in adults.

##### Organophosphates

The systematic analysis of the studies in this section is displayed in Supplementary Tables 4a,b. A total of 17 preclinical studies were included in the present systematic review. From these, 4 (24%) were labeled as high, 7 (41%) as medium-high and 6 (35%) as medium quality, two of them with low to very low sample size. At the same time, 13 studies with humans were included, 2 of them (15%) classified as high, 4 (31%) as medium-high, 2 (15%) as medium, 4 (31%) as medium-low and 1 (8%) as low quality, all of them with acceptable sample size.

##### Impulsivity and OPs

At a preclinical level, 8 studies analyzed the influences of OP exposure on impulsivity, both choice and action subtypes. From these, 5 (63%) were labeled as medium-high (Cardona et al., 2011; López-Granero et al., 2013, 2014; Terry et al., 2014; Peris-Sampedro et al., 2016) and the remaining 3 (37%) as medium quality (Cardona et al., 2006; Middlemore-Risher et al., 2010; Montes de Oca et al., 2013).

Cardona et al. (2006) found increased long-term impulsive choice after acute exposure to high doses of CPF in adult rats. The high compulsive rats (split by SIP) had larger rates of impulsivity and altered decision-making. Follow up study also found both short and longer-term increased impulsive choice in high-compulsive CPF-exposed rats (Cardona et al., 2011). However, this same high acute dose was not able to induce either short or long-term impulsive performance in wistar rats, regardless of whether parathion (15 mg/kg) nor Diisopropylfluorophosphate (1.5 mg/kg) OP compounds (López-Granero et al., 2014) were used. However, López-Granero et al. (2013) found impaired impulsive choice associated with low CPF chronic dietary exposure for 31 consecutive weeks, followed by an increase in AChE read-trough variant expression in exposed animals.

Beyond choice, impulsive action was also studied in relation with OP exposure. On this way, Middlemore-Risher et al. (2010) found that chronic moderate doses of CPF for 14- or 30-days increased premature responding (5-CSRTT), with higher rates in the 30-day condition. Further, washout periods revealed even greater impulsive responding in exposed rats in general. Terry et al. (2014) used the OP agent Diisopropylfluorophosphate for 30 consecutive days in adult wistar rats. Authors found that increments in the inter trial interval generally increased premature responding. Interestingly, this effect was attenuated in exposed animals compared control rats during exposure, with the opposite effect during washout periods. However, such effects of OP compounds on impulsive action have not always been observed (Montes de Oca et al., 2013; Peris-Sampedro et al., 2016).

Based on human studies, 9 works analyzed impulsive outcomes in relation with OP exposure. From these, 2 (22%) were classified as high (Marks et al., 2010; Wesseling et al., 2010), 3 (33%) as medium-high (Ruckart et al., 2004; Kofman et al., 2006; Fortenberry et al., 2014), 1 (11%) as medium (Suarez-Lopez et al., 2017) and 3 (33%) as medium-low quality (Zhang et al., 2009; Mackenzie Ross et al., 2010; Joo and Roh, 2016).

Particularly with exposure in children, symptoms derived from high doses of OP agents were linked to increased motor impulsivity assessed with the NEPSY (Kofman et al., 2006). Other authors found alterations in parental-reported impulsive control (the Pediatric Environmental Neurobehavioral Battery and the Personality Inventory for Children) in exposed children (Ruckart et al., 2004). Furthermore, Suarez-Lopez et al. (2017) recently reported poorer inhibitory control (NEPSY-II) in children who suffered environmental exposure during specific months of the year (e.g., Mother's Day flowers harvest). Authors observed than these effects increased when approaching the critical time of exposure, with girls showing the worst performance. Added to this, ADHD symptomatology was linked to OP metabolite levels at gestational and, in lesser degree, postnatal urinary samples (Marks et al., 2010), eminently with the attentional dimension. Finally, other studies also found no relation between gestational exposure and ADHD symptomatology in children (Fortenberry et al., 2014).

Studies conducted in the adult population linked OP exposure to both impulsivity traits, with special emphasis on suicidal behaviors. Suicide ideation is a complex behavior in which impulsivity traits play an important role. Zhang et al. (2009) found that people who suffered chronic exposure (storage of OP compounds at home) increased the percentage of suicidal ideation in comparison with non-storers. Similarly, both self-reported depressive symptoms and suicidal traits were associated with OP exposure, but not with Carbamate agents (Wesseling et al., 2010). Moreover, higher number of OP exposure symptoms have recently been linked to suicide attempts, impulsivity (BIS), and aggressive behaviors (Lyu et al., 2018, published as letter to editor). Finally, recent study conducted by Joo and Roh (2016) found larger rates of suicidal ideation and depression in farmers, although specific xenobiotic agents were not discussed.

##### Compulsivity and OPs

A total of 15 preclinical studies analyzed compulsive outcomes following OP exposure. From these, 4 (27%) were categorized as high (McDonald et al., 1988; Maurissen et al., 2000; Sánchez-Santed et al., 2004; Timofeeva et al., 2008), 5 (33%) as medium-high (Cardona et al., 2011; Terry et al., 2014; Savy et al., 2015, 2018; Peris-Sampedro et al., 2016) and 6 (40%) as medium quality (Raffaele et al., 1990; Cardona et al., 2006; Middlemore-Risher et al., 2010; Chen et al., 2012; Terry et al., 2012; Montes de Oca et al., 2013).

Cardona et al. (2006) found that the single, high dose of CPF previously indicated also increased compulsive behavior in high drinking rats compared with a high drinking control group. A similar observation was reported in follow up studies, although this increased compulsive trait was found irrespective of high/low drinker status (Cardona et al., 2011), apparently with an important GABAergic influence. Further, the high, acute dose of CPF previously described in Cardona's studies was also linked to a long-term increased perseveration (5-CSRTT) in rats (Montes de Oca et al., 2013). Such perseverative responding rates were ameliorated following amphetamine challenge, thus indicating an important dopaminergic role on its regulation. Authors also found increased dopamine tone in the hippocampus but not the striatum, as well as decreased levels of both GABA and glutamate in the striatum.

Similarly, increased perseverative responses following CPF exposure were found in the previously described Middlemore-Risher's study, but only during washout periods in the longer exposure protocol and subtle decreases during exposure. Like what was found with impulsive responding, Peris-Sampedro et al. (2016) reported that CPF also blocked the basal higher perseverative responding rate of the APOE-4 mice. Interestingly, Terry et al. (2014) did not find altered perseverative responding but inflexibility during time-out periods following Diisopropylfluorophosphate exposure. These authors also found this pattern in the reversal stage of a water maze paradigm following adolescent exposure (Terry et al., 2012), both studied conducted with wistar rats.

In other terms, MBT has been scarcely studied in relation to OP exposure. In this regard, Savy et al. (2015) found that sub-chronic 5-day exposure to low doses of both CPF and Diazinon decreased compulsive-like behavior (MBT) in adult rats for a further week in the case of Diazinon, which is related to 5-HT transporter downregulation in both the frontal cortex and hippocampus. Interestingly, Diazinon also produced similar effects at longer exposure periods (Savy et al., 2018).

Following this, early studies also found a decreased switching capacity (increased perseveration) linked to exposure to other types of OP such as Diisopropylfluorophosphate and Disulfoton in alternation tasks, along with a possible link with decreased muscarinic binding at different brain areas (McDonald et al., 1988). In a similar vein, Chen et al. (2012) found altered DAT performance, with a strong increase of lose-shift errors in male mice exposed during gestation, particularly at longer delays and along with decreased cells number at both hippocampal and frontal structures. However, these types of effects have not been systematically observed following low, sub-chronic exposure during development (Maurissen et al., 2000; Timofeeva et al., 2008) or high, acute doses of CPF during adulthood (also Paraoxon) in DAT (Sánchez-Santed et al., 2004).

Finally, to the best of our knowledge, studies of compulsivity traits related to OP exposure in human population are sparse. A total of 5 studies were included, 1 (20%) categorized as medium-high (Ismail et al., 2017), another (20%) as medium (Mittal et al., 2011), 2 (40%) as medium-low (Mackenzie Ross et al., 2010; Rohlman et al., 2016) and 1 (20%) as low quality (Sánchez Lizardi et al., 2008).

In this regard, higher rates of perseveration assessed by WSCT were positively correlated with OP metabolite levels in 7-year old children (Sánchez Lizardi et al., 2008). In later childhood, Mackenzie Ross et al. (2010) found altered flexibility (CALCAP choice, trails B and Stroop tests) in the exposed group compared with a non-exposed group. Furthermore, TMT alterations were also linked to postnatal OP exposure, albeit authors found no effects in reversal tasks (Rohlman et al., 2016). This was also observed in both TMT and verbal fluency test following high accidental exposure (Mittal et al., 2011). Authors linked this to a general alteration of blood flow patterns in exposed participants, particularly affecting males in the occipital areas of the right hemisphere. Furthermore, Trail Making Test alterations following pesticide exposure have not been systematically found (Ismail et al., 2017).

## Discussion

The present manuscript summarizes the most important empirical works published in English and focused on the relationships between the exposure to 4 xenobiotic compounds commonly found in human environments (Pb, MeHg, PCBs, and OPs) with the different subtypes of impulsive (choice and action) and compulsive (perseveration) behaviors. This is, to the best of our knowledge, the first time that these agents are systematically analyzed in relation to their specific properties in the regulation of impulsive and compulsive sub-traits, both human and non-human research and into a single manuscript.

Most of the systematic and classical reviews focused on ADHD and ASD, with an important role of impulsive traits (de Cock et al., 2012; Polanska et al., 2013; Daneshparvar et al., 2016). In relation to OP and other pesticides, different reviews on suicide behaviors were also done (London et al., 2005; Freire and Koifman, 2013). Briefly, these studies globally concluded that the exposure to the different agents is linked to increased ADHD symptomatology, also hyperactive/impulsive type. However, attentional alterations were more commonly found and MeHg influences seems to be weaker and less studied, with lead as the best analyzed by far. Compulsive traits have been basically not explored and arranged in relation to the exposure to these agents.

### Main Findings

#### Impulsivity and Xenobiotic Compounds

The systematic analysis of the most important empirical studies on the different neurotoxicological agents shows similar effects amongst them. On this way, the strong point throughout all compounds is a clear link between both gestational and postnatal exposure with increased impulsive action as well as general hyperactivation.

Pb was early linked to specific increase of both activity and improper response inhibition. Most of the preclinical studies clearly found this negative relation between exposure and impulsive action. Larger impulsive rates were systematically found throughout the different quality ranges, with only counted exceptions (Angell and Weiss, 1982 -post weaning exposed; Rice and Gilbert, 1985). This hyperactivity and motor impulsive patterns were early described by non-human primates works in Rice's studies (i.e., Rice et al., 1979; Rice, 1992) and researching with murines represented by Cory-Slechta's works (i.e., Cory-Slechta and Thompson, 1979; Cory-Slechta et al., 1981). The use of specifically designed fixed intervals/ratios, progressive ratios and DRL schedules following low to middle chronic exposures to Pb during postnatal development systematically triggered a long-term prepotent responding pattern. However, most of the preclinical studies were done with low to very low sample size, strongly limiting further generalizations.

Interestingly, this strong relation between postnatal exposure rates and impulsive action increase was systematically found in human studies, both children and young adults (i.e., Nicolescu et al., 2010). Furthermore, all human studies analyzed here found significant increased rates of impulsive behavior in relation with exposure degree, thus methodological quality should not be an important factor on this association. This can also be found in some pervious reviews on ADHD patients such as Daneshparvar et al. (2016), albeit attention deficits were stronger associated with Pb exposure.

Related to MeHg, increased impulsivity rates in preclinical models have been systematically observed independently the quality of the study, with some exceptions at the medium-high range (i.e., Sable et al., 2009). Both gestational and early postnatal exposure increased impulsive action rates both non-primate humans and, eminently, rats and mice models. In this case, few studies found an additive effect of gestational-early postnatal continuous exposure to MeHg in mismatched performance in different type of operant schedules (i.e., Paletz et al., 2006; Reed and Newland, 2007).

Clinical research on MeHg influences on impulsive action triggered inconclusive, contrary results. Only in counted cases, authors replicated the increased impulsivity found in preclinical studies following gestational (i.e., Stewart et al., 2006) and postnatal (i.e., Yokoo et al., 2003; Lee et al., 2018). However, many works had negative results on this issue (i.e., Stewart et al., 2003, 2005). Attending to their relative quality, both high classified studies (Ethier et al., 2015; van Wijngaarden et al., 2017) did find no significant influences of MeHg exposure on impulsive traits and only 2 out of 8 medium-high quality studies (Yokoo et al., 2003; Stewart et al., 2006) observed these influences. This inconsistence and lack of enough empirical research was also declared in previous-made reviews focused on ADHD symptomatology (i.e., Polanska et al., 2013).

Albeit confusing, these data are not unexpected, due to one of the most common exposure source for humans comes from fish and crustaceous consumption along with other industrial origins such as burning coal. Thus, the negative effects on cognition derived from MeHg exposure in humans is probably masked by other “positive for health” molecules present into fish, disconnecting both clinical and preclinical results. Some authors have proposed than some of these molecular targets could be the Omega-3 fatty acids present into some fish varieties, Selenium levels found into the seafood (eminently for inorganic mercury) and/or vitamin E interactions. However, all these candidate chemicals showed contradictory (Omega-3 fatty acids) or insufficient empirical support (Se and vitamin E) (Mergler et al., 2007). Interestingly, Omega-3 fatty acids diet supplementation has been linked to reduced cognitive and motor impulsivity (BIS) (Conklin et al., 2007) as well as improved impulsive profile in ADHD children (Derbyshire, 2017). Furthermore, these effects could be mediated by the regulation of the 5-HT system (Patrick and Ames, 2015). Attending to the data summarized here, increased impulsive rates following MeHg exposure in humans has not been systematically evidenced.

Furthermore, several studies have found increased premature responding pattern as well as general hyperactivation following PCB exposure in different paradigms (i.e., Lilienthal et al., 1990; Rice, 1997; Berger et al., 2001). Attending to quality, most of the high and medium-high quality works shows this profile with one exception at each category (Rice and Hayward, 1998, 1999b, respectively). Both gestational and early postnatal developmental stages are especially sensitive to PCBs, but postnatal influence has stronger empirical support attending to 8 out of 15 are studies with exclusive postnatal exposure and the rest are continuous gestational/postnatal exposure.

These preclinical studies have their translation meaning in different works on humans where increased commission errors and general impaired response inhibition were linked to both gestational and postnatal exposure (Stewart et al., 2003, 2005, 2006), also attentional performance (i.e., Sagiv et al., 2010, 2012). When analyzed by methodological quality, the only high-rated study did not find this relation following both gestational and postnatal exposure, but altered attentional performance was found (Either et al., 2015). Furthermore, 5 out of the 13 studies categorized as medium quality found non to little relation between exposure rates and impulsive outcomes, without developmental exposure stage influence. Interesting, some cohorts like Stewart's and Jacobson's are consistent in this negative relationship. This contradictory information could be due to strong differences in population, assessment tools and/or PCB congener.

Added to this, some works linked CPF and other OP agent's exposure with increased impulsive choice and action by premature responding (i.e., Cardona et al., 2006; Middlemore-Risher et al., 2010; Terry et al., 2014). However, an important disparity exists with no effects in some other works (i.e., Montes de Oca et al., 2013). Nevertheless, this could be explained by the inconsistency between researching groups by doing completely different exposure patterns, both in terms of dosage and time of exposure.

Attending to quality level, impulsive choice rates have been systematically increased by OP exposure in both medium-high (Cardona et al., 2011; López-Granero et al., 2013) and medium quality (Cardona et al., 2006), albeit exceptions have been also noticed (López-Granero et al., 2014). In relation with impulsive action, only one medium-high cataloged study (Terry et al., 2014) found decreased rates during exposure but increase premature responding during wash-out periods. This was also been found in lower-rated studies such as (Middlemore-Risher et al., 2010). That is to say, the apparent consistency in the increased discounting following OP exposure contrasts with the contradictory information from motor impulsivity.

Following this, increased impulsive rates in children have been linked to OP levels, both by neurocognitive task and questionnaire-based protocols (i.e., Ruckart et al., 2004; Kofman et al., 2006). Furthermore, such agents have been linked to suicide behaviors as well as suicidal ideation (i.e., Zhang et al., 2009; Wesseling et al., 2010), as previous summarized in other reviews (i.e., London et al., 2005). Albeit depressive and other mood mismatching could be at the basis of this extreme behavioral pattern, impulsive reasoning and execution seems to be an important variable in its development. Attending to the preclinical empirical information explained here, this idea gains support. Quality assessment demonstrated that from the 2 high quality studies, only one (Wesseling et al., 2010) found this negative relationship but specifically on suicide ideation, while this is slightly increased in medium-high studies (2 out of 3, 67%) and in all the studies lower labeled (4). Thus, the apparent clear link between OP exposure and impulsive traits in humans is moderated when methodological procedures are improved.

#### Compulsivity and Xenobiotic Compounds

Several studies have systematically associated Pb exposure with a perseverative responding pattern following multitude of reversal tasks, both spatial and non-spatial, both relevant and irrelevant associated stimuli (i.e., Bushnell and Bowman, 1979a; Newland et al., 1994). This inflexibility or insensitivity to changing contingencies was also found when changing rules happened even in a continuous fashion (i.e., DAT paradigm). Like previously found for impulsive action, postnatal exposure has been systematically associated with such impairments, albeit some works defended the stronger influence of concurrent over early exposure (i.e., Gilbert and Rice, 1987).

However, when deeply study the quality of each methodology, 2 out of the 4 better classified studies did not find this relation (Hastings et al., 1984; Garavan et al., 2000), and the other two included glutamatergic drugs into their analysis with different results (Cohn and Cory-Slechta, 1993, 1994). However, this negative association is systematically observed throughout the remaining quality categories, with some exceptions (Levin and Bowman, 1988).

Some studies such as Hilson and Strupp (1997), Rice (1985) or Garavan et al. (2000) discriminate between pure perseveration and altered learning after a change of contingencies. They concluded that the alteration at late but not early stages in a reversal condition could be more associated with a learning disturbance than with a compulsive-like pattern. This kind of specification when studying compulsive traits is essential in order to control possible learning, higher-order influences on our conclusions. This could be also added to the common lack of separation between hyperactivity and impulsive action in animal models.

Although generalization is limited attending to the number of the studies done, increased rates of compulsive traits have been found throughout all the four human studies analyzed, independently of their relative quality (Stiles and Bellinger, 1992; Evans et al., 1994; Surkan et al., 2007; Nicolescu et al., 2010).

Similar data were found with Pb exposure were linked to MeHg; developmental exposure to MeHg decreases sensitivity to changing contingencies as well as a strengthens perseveration by an inflexible pattern in exposed animals, both with and without spatial component (i.e., Reed et al., 2006). This happened it does not matter whether it took place in reversal or extinction conditions. Otherwise, DAT and other alternation tasks showed contradictory results. Attending to quality influence, the 3 highest classified works found opposite results in the relation of MeHg exposure with perseveration and inflexibility rates, with two studies showing negative influences (Doré et al., 2001; Boomhower and Newland, 2017) and one without this effect (Goulet et al., 2003). Meanwhile, medium-high studies showed a clear negative influence of MeHg most cases (83%) with counted exceptions (Goldey et al., 1994). All this without an apparent dosage or developmental stage influence.

This apparent consistency contrasts with the almost non-existent empirical work in humans, where from 3 works, 2 found significant negative influences of postnatal MeHg exposure (Philibert et al., 2008; van Wijngaarden et al., 2017). On this way, the presumably negative effects of MeHg exposure on compulsive behavior have not enough empirical support in humans.

Different preclinical and clinical studies have linked PCB exposure with increased perseverative and inflexible patterns. However, only one out of three high and two out of 7 medium-high quality preclinical studies (29%) have shown this effect (Holene et al., 1998; Sable et al., 2006). The lowest classified studies showed this negative effect on perseveration and flexibility (Rice and Hayward, 1997; Widholm et al., 2001). This pattern was found in multiple tasks with reversal stages with or without spatial components, external control stimuli (relevant or irrelevant) influences and in DAT-like tests or extinction phases. However, most of the analyzed studies (7 out of 11) did not find any effect on compulsive perseveration or, in some cases, did not specifically describe perseverative responding in DAT.

All this must be added to the lack of empirical study of the influences of PCB exposure on human cognitive flexibility and perseveration, attending to the limited, contradictory results that currently exist (Jacobson and Jacobson, 2003; Fimm et al., 2017). This is presumably due to the important methodological differences amongst studies. Taking all together, the increase of compulsive behaviors in animals is, at least, not robust and in humans basically not explored.

Like this, CPF and other OP compounds increased perseverative responding in different paradigms such as 5C-SRTT or SIP, where susceptible basal compulsive animals were even more inflexible following CPF exposure (i.e., Cardona et al., 2011). Only 1 out of the 4 high quality studies found increased rates of perseverative behavior (McDonald et al., 1988). Otherwise, from the 5 medium-high quality works, two of the studies showed increased rates (Cardona et al., 2011; Terry et al., 2014) and other two reduced rates (Savy et al., 2015, 2018). However, it seems that OP alters specific components of compulsivity more related to perseveration (SIP, 5C-SRTT) than other paradigms strongly affected by other variables such as anxiety and working memory (MBT and DAT).

Finally, the influences of the different OP compounds exposure on human have been little studied, with only a few representatives which exert, in some cases, opposite information. The higher classified study (Ismail et al., 2017) did not show such influence of OP exposure on compulsive traits, meanwhile the remaining (4), lower-categorized works found this negative influences with different assessment methodologies.

### Neurodevelopmental Exposure Stages and Impulsive/Compulsive Traits

From all the studies summarized here, most of them exposed animals or took biological samples during development. From these, the ≈50%, 100%, ≈80% and the 18% of the preclinical studies on Pb, MeHg, PCBs, and OPs started their exposure protocol at a pre-gestational, gestational or early postnatal stage. Otherwise, all the clinical studies of Pb, MeHg, and PCBs exposure worked with children and used gestational and/or postnatal exposure measures. The exception of the OPs in the clinical field (≈40%) is reasonable attending to their main use as pesticides, being applicators the main population of exposure. Otherwise, the low developmental rate in preclinical research is surprising as developmental OP exposure to low or very low doses is the most research field in the neurotoxicology from, at least, the early 2000s' (i.e., Slotkin and colleagues' extensive studies).

### Limitations of the Present Systematic Review

The present manuscript has faced several difficulties based on 3 different aspects: (1) searching strategy, (2) evolution of impulsivity/compulsivity constructs definition, (3) standardization and specificities of some of the behavioral paradigms.

Briefly, Boolean searching strategy was found inefficient in order to achieve all the titles we included into the review. In fact, an important amount of studies (some of them with a capital importance for our scope) were included thanks to the referenced works by the initially analyzed manuscripts. Attending to this, we cannot discard some important studies could be out of the analysis. This was also related to the misused of standardized paradigms but different operant manipulations, eminently in early studies. Some studies did not explicitly discuss their behavioral outcomes in terms of impulsivity or compulsivity, but a general inhibitory control functioning or, in some cases, defining a clear impulsive pattern with attentional/hyperactivation mismatching. The lack of these concepts (impulsivity, compulsivity, DAT, 5C-SRTT, amongst others) make the idea of access to all the published studies a hard issue. Finally, some paradigms are known to have an important component from other cognitive functions such as working memory (DAT), attention (TMT and Stroop) and anxiety (MBT) and the real meaning of the main outcomes still under intense discussion (Moritz et al., 2009; Thomas et al., 2009; Angoa-Pérez et al., 2013). In this regard, some of the outputs discussed here as compulsivity could be discussed in other terms.

## Conclusions and Future Guidelines

However, the present systematic review lets to conclude that Pb, MeHg, PCBs, and OPs have the capacity to induce specific alterations in mammalian (mice, rats, monkeys, and humans) in their capacity to control improper behaviors, behold preponderant responses (motor impulsivity), and adapt to new contingencies (inflexibility/perseveration). However, there are strong limitations which are relatively shared amongst such compounds: (1) the important empirical support of the increased impulsive action and compulsive perseveration following Pb exposure is weakened by the improper sample size used by an important part of the preclinical studies. (2) The lack of parallel analysis of motor activity and/or motivation in preclinical models do not let us to discard these factors from the main variables (impulsivity or simply hyperactivation?). (3) The substantial empirical support in relation to the effects of MeHg exposure in preclinical models contrasts with the contradictory findings from human studies, probably due to the exposure source (i.e., positive effects on health from other substances present in fish such as omega-3 fatty acids). (4) Impulsive choice has rarely been explored both animals and humans. (5) Compulsive behavior study is almost non-existent and unspecific in humans. 6) There is a general lack of linking between human studies and preclinical models. (7) There is an apparent lack of the influences of developmental exposure to OP on impulsive and compulsive traits.

To our criteria, it is essential to implement delayed discounting-like tasks and other related paradigms with decision-making as well as risk taking outcomes in preclinical works following those different compounds exposure. Motor and motivational parallel outcomes must be also analyzed in order to avoid uncontrolled influences on the main impulsive/compulsive behaviors in preclinical studies. It is also surprising the little number of developmental OP exposure effects on impulsive and compulsive behaviors, attending to the vast research on both gestational and postnatal exposure in relation with other cognitive functions. Specific studies on this issue should be conducted. Finally, human studies should take advantage on compulsive traits, both by questionnaire-based and neurocognitive tasks in relation to the level of exposure, following similar and adapted tasks previously observed as very sensitive in non-primate humans.

## Author Contributions

All authors contributed to the present manuscript. CP-F made the searching protocol, analyzed the different studies, wrote the first version of the manuscript and applied all the necessary changes. PF and FS-S supervised the whole procedure as well as re-structured and improved general lines until get the current status of the manuscript.

### Conflict of Interest Statement

The authors declare that the research was conducted in the absence of any commercial or financial relationships that could be construed as a potential conflict of interest.
