# Supplementary material for: A Systematic Review on the Influences of Neurotoxicological Xenobiotic Compounds on Inhibitory Control
Source: Front Behav Neurosci. 2019 Jul 4;13:139. doi: 10.3389/fnbeh.2019.00139 (PMC6620897; doi:10.3389/fnbeh.2019.00139)
Supplement: Supplementary file 1 [file Data_Sheet_1.PDF]

Age & Sex & Strain	Dose & Exposure time & route	Exposure control	Behavioral test/Questionnaires	Behavioral/Pharmacological/Physiological outcomes	Reference	Quality Index
PND90<br>M 100%<br>LE	545-1090 ppm from gestation-onwards	Pb levels from blood	Spatial discrimination reversal task; Simultaneous visual discrimination + Delay	Compulsivity, inflexibility- Exposed = CNT	Hastings et al., 1984	H+
PND55<br>M 100%<br>LE	50-250 ppm from PND21-onwards	Pb levels from blood	Multiple repeated acquisition and performance	Compulsivity, perseveration- Exposed + MK-801 < CNT + MK-801	Cohn & Cory-Slechta, 1993	H+
PND55<br>M 100%<br>LE	50-250 ppm from PND21-onwards	Pb levels from blood	Multiple repeated acquisition and performance	Compulsivity, perseveration- Exposed + MK-801 > CNT + MK-801	Cohn & Cory-Slechta, 1994	H-
PND53<br>M 0%<br>LE	300 ppm<br>GD0-PND30<br>300 or 600 ppm from PND1-30	Pb levels from blood	Two-choice discrimination olfactory non-spatial reversal task	Compulsivity, inflexibility- Exposed ≈ CNT // Learning/associative- Exposed < CNT	Garavan et al., 2000	H?
Infants-Young<br>M ≈50%<br>RM	≈ 0.287-0.880 mg/kg/day PNW3 for 1 year.	Pb levels from blood	Spatial and non-spatial (color & size) discrimination reversal tests; Adapted Sackett Self-Selection;	Compulsivity, inflexibility- High exposed > CNT // Learning- Low exposed < CNT // Motivation/overtraining- Exposed = CNT	Bushell and Bowman, 1979a	MH-
PND55<br>M 100%<br>SD	100 or 300 ppm<br>From PND≈21-onwards	Pb levels from Brain, liver and kidney	DRL-task with tone facilitation	Impulsive action- Exposed animals > CNT; no effects of external auditory stimulus for help exposed animals	Cory-Slechta et al., 1981	MH-
5-6 y.o.<br>M 33%<br>MF	1.5 mg/kg/day<br>From PND1-onwards;<br>From PND1-PND400;<br>From PND300-onwards.	Pb levels from blood	Non-spatial (form and color) discrimination<br>reversal tasks with relevant and irrelevant cues	Compulsivity, inflexibility- Exposed from birth > CNT in general (early reversals); Exposed from PND300 > CNT in both form and color discrimination (with irrelevant cues); PND1-400 = CNT // Learning- Exposed from birth < CNT when irrelevant color cues.	Rice & Gilbert, 1990a	MH+
Adulthood<br>M 0%<br>LE	75-300 ppm<br>from early gestation<br>Mother	Pb levels from blood	Olfactory discrimination test with reversal; Extra dimensional shift task	Compulsivity, inflexibility- High exposed ≈ rest (reversal) // Compulsivity, inflexibility with attentional shifting- exposed > CNT (EDS) // Learning/associative- High exposed < rest	Hilson & Strupp, 1997	MH?
PNW9<br>M 100%<br>LE	5.27 mmol/L/day<br>From PND1-21	Pb levels from blood and brain	Two-choice discrimination olfactory non-spatial reversal task	Compulsivity, inflexibility- Exposed > CNT //Learning- Exposed < CNT, worsened by poor fatty acid diet.	Lim et al., 2005	MH?
PND55<br>M 100%<br>SD	50, 300-1000 ppm from PND≈21-PND91	Pb levels from kidney and liver.	Fixed interval schedule of reinforcement	Impulsive action- Exposed 50 & 300 > CNT & high exposed	Cory-Slechta & Thompson, 1979	M?
3-4 y.o.<br>M 46%<br>MF	50 or 100 ug/kg/day, 5 days per week from PND1 to onwards.	Pb levels from blood	DRL	Impulsive action- Both exposed= CNT // Learning- Exposed<CNT	Rice & Gilbert, 1985	M-
7-9 y.o.<br>M (N.I.)	Variable dosage (High pulses) PNW3 for 1 year	Pb levels from blood	DAT (Acquisition)	Compulsive, perseveration- Exposed > CNT	Levin & Bowman, 1986	M?

RM						
9 y.o.<br>M (N.I.)<br>RM	≈ 0.287-0.880-1 mg/kg/day<br>PNW3 for 1 year.	Pb levels from blood	DAT	Compulsive, perseveration- Exposed = CNT	Levin & Bowman, 1988	M-
7-8 y.o.<br>M 46.2%<br>MF	50-100ug/kg/day 5 days/week<br>From PND1 to onwards	Pb levels from blood	DAT	Compulsivity, perseveration- Exposed (eminently high) > CNT (longer delays) // Learning- Exposed < CNT	Rice & Kaprinski, 1988	M-
5-6 y.o.<br>M (N.I.)<br>SM	44-79ug/dL<br>From GW5/8.5	Pb levels from brain & blood	Multiple RI-RI transitory schedule of reinforcement tasks	Compulsivity, inflexibility- Exposed > CNT	Newland et al., 1994	M-
PND40<br>M 100%<br>LE	50-150 ppm from PND21-onwards	Pb levels from blood	Fixed interval schedule of reinforcement	Impulsive action- Exposed + DA drugs < CNT + DA drugs	Cory-Slechta et al., 1996	M+
PND60<br>M 100%<br>LE	50 or 150ppm from PND21 to onwards	Pb levels from blood	Multiple FR waiting-forward schedule of reinforcement	Impulsive action- Exposed (stronger in high exposed) > CNT	Brockel & Cory-Slechta., 1998	M+
PND≈50<br>M 100%<br>LE	50 or 500 ppm<br>From PND≈21-onwards	Pb levels from blood	Fixed interval schedule of reinforcement	Impulsive action- High exposed > CNT; summative effect with DA antagonism (N-ethoxycarbonyl-2-ethoxy- 1,2-dihyroquinoline) // Several dopamine system implications	Cory-Slechta et al., 1998	M+
PND≈50<br>M 100%<br>LE	50 or 500 ppm<br>From PND≈21-onwards	Pb levels from blood	Fixed interval schedule of reinforcement	Impulsive action- High exposed > CNT. Compulsivity, perseveration- High exposed > CNT (later sessions) // Several dopamine system implications	Cory-Slechta et al., 2002	M+
PND62<br>M 0%<br>LE	300+300-300+20 ppm from PND1-30	Pb levels from blood & Brain	Visual discrimination task + delay; Sustained/selective attention tasks	Impulsive action- High exposed > CNT // Quelation moderated such effects, eminently in low exposed // Learning- Exposed < CNT	Stangle et al., 2006	M+
PNW7<br>M 0%<br>Mice H67D Hfe	1.6-2.5 mg/kg/day from PNW3-onwards	Pb levels from brain & liver	MBT; Nestled shredding test	Compulsivity- Exposed > CNT // Hfe mutation blocked such differences // TH & dopamine transporter expression- Exposed > CNT	Chang et al., 2014	M?
4 y.o.<br>M ≈50%<br>RM	≈ 0.287-0.880 mg/kg/day from PNW3 for 1 year.	Pb levels from blood	Spatial discrimination reversal tests with relevant & irrelevant cues	Compulsivity, inflexibility- Both exposed > CNT // Learning- High exposed < CNT	Bushell and Bowman, 1979b	ML-
2.5-3 y.o.<br>M 50%<br>MF	500ug/kg/day from PND1-onwards	Pb levels from blood	Multiple fixed interval schedule of reinforcement	Impulsive action- Exposed > CNT // Motricity- Exposed = CNT // No amphetamine effects	Rice et al., 1979	ML-
PND58<br>M 50%<br>LE	PND3-onwards or PND21-onwards.	Pb levels from brain and blood	Multiple fixed interval/ratio with Time outs.	Impulsive action- Post-weaning exposed < CNT; Pre-weaning exposed > CNT (slightly);	Angell & Weiss, 1982	ML+
PND50<br>M 100%<br>LE	25ppm from ≈PND21-onwards	Pb levels from blood & brain	Fixed interval schedule of reinforcement/VT-90sec	Impulsive action- Exposed > CNT (early stages)	Cory-Slechta et al., 1985	ML+

3-4 y.o.<br>M 46%<br>MF	50-100 ug/kg/day, 5 days per week from PND1 to onwards.	Pb levels from blood	Non-spatial (color and form) discrimination reversal tasks with relevant & irrelevant cues	Compulsivity, inflexibility- High exposed > rest (general and form) // Attentional & associative- Low exposed < CNT	Rice 1985	ML?
9-10 y.o.<br>M 46%<br>MF	50 or 100 ug/kg/day, 5 days per week from PND1 to onwards	Pb levels from blood	Spatial discrimination reversal tasks with both relevant or irrelevant (color or form) cues	Compulsivity, inflexibility- Exposed > CNT when irrelevant stimuli were displayed // Learning- High exposed < CNT (irrelevant form cues)	Gilbert & Rice, 1987	ML-
7-9 y.o.<br>M (N.I.)<br>RM	Variable dosage (High pulses) PNW3 for 1 year	Pb levels from blood	DAT (Post-acquisition)	Chronic dopamine agonist (L-Dopa) partially blocked Pb alterations	Levin et al., 1987	ML-
7-8 y.o.<br>M 33%<br>MF	1.5mg/kg/day<br>Birth-onwards; Birth-PND400; PND300-onwards.	Pb levels from blood	Spatial discrimination reversal tasks with relevant and irrelevant (new and familiar) cues	Compulsivity, inflexibility- Exposed from birth > CNT in general // all groups were impaired with new irrelevant form cues in general, exposed from birth at early reversals; Exposed from birth were impaired with familiar irrelevant cues // Learning- Exposed = CNT	Rice 1990	ML+
6-7 y.o.<br>M (N.I.)<br>MF	1.5 mg/kg/day<br>From PND1-onwards;<br>From PND1-PND400;<br>From PND300-onwards.	Pb levels from blood	DAT	Compulsivity, perseveration- Exposed > CNT (longer delays)	Rice & Gilbert, 1990b	ML+
5-6 m.o. - 10 y.o.<br>M 50%<br>MF	2 mg/kg/day<br>From PND1-onwards	Pb levels from blood	Non-spatial discrimination reversal tasks with relevant & irrelevant cues (form & color); DRL; DAT; Multiple fixed interval/ratio schedule of reinforcement	Discrimination task. Compulsivity, inflexibility- Exposed > CNT (infants); when irrelevant form cues Exposed > CNT (juvenile) // DRL task. Impulsive action- Exposed > CNT (adults) //DAT. Compulsivity, perseveration- Exposed = CNT (adults) //Learning- Exposed= CNT in all ages and tasks.	Rice 1992	ML-
