# Supplementary material for: A Systematic Review on the Influences of Neurotoxicological Xenobiotic Compounds on Inhibitory Control
Source: Front Behav Neurosci. 2019 Jul 4;13:139. doi: 10.3389/fnbeh.2019.00139 (PMC6620897; doi:10.3389/fnbeh.2019.00139)
Supplement: Supplementary file 2 [file Data_Sheet_2.PDF]

Age & Sex	Dose & Exposure time	Exposure control	Behavioral test/Questionnaires	Behavioral/Pharmacological/Physiological outcomes	Reference	Quality Index
11.2 y.o. M 67%	Pre and postnatal exposure	Pb levels from umbilical cord and child's blood	Visuospatial attention-shift paradigm	P.C. gestational Pb levels_Impulsive action	Ethier et al., 2015	H+
12.4 y.o. M 68%	Postnatal exposure	Pb levels from child's blood	DSM-IV; Behavior disorders & symptoms	P.C. Post-natal Pb levels_Impulsive/hyperactive ADHD type symptoms (eminently parents-referred)	Nigg et al., 2010	H+
10 y.o. M (N.I.)	Pre and postnatal exposure	Pb levels from umbilical cord and child's blood	WCST; California Verbal Learning Test for Children	P.C. Pb24 levels_Compulsivity, perseveration (CVLT-C) // N.C. Pb12_ Compulsivity, perseveration (CVLT-C) // P.C. Pb57 levels_Compulsivity, perseveration (WSCT)// P.C. Pb120_Compulsivity, perseveration	Stiles et al., 1993	MH+
4.5 y.o. 45 %	Pre and postnatal exposure	Pb levels from umbilical cord and child's blood	Michigan Catch-the-Cat Test (CPT)	P.C. Pb levels_Impulsive action (early block) // N.C. Corpus Callosum volume (Splenium)_Impulsivity rates.	Stewart et al., 2003	MH+
9.5 y.o. M (N.I.)	Pre and postnatal exposure	Pb levels from umbilical cord and child's blood	DRL	N.C. Postnatal Pb_ money earned	Stewart et al., 2006	MH+
8 y.o M ≈46%	Postnatal exposure	Pb levels from child's blood	Stroop Test; WCST; TMT	Compulsivity, perseveration- >5ug/dL > <5ug/dL Children	Surkan et al., 2007	MH+
9.9 y.o. M 51%	Postnatal exposure	Pb levels from blood	Test battery for attention performance of children (alertness, GNGT, distract-ability and flexibility subtests); DSM-IV	P.C. Postnatal Pb levels_Impulsivity (alertness & action & parents referred) and compulsivity (inflexibility).	Nicolescu et al., 2010	MH+
5.4 y.o. M 43.6%	Pre and postnatal exposure	Pb levels from umbilical cord and child's blood	Infant Behavior Rating Scale	P.C. postnatal Pb levels_Impulsivity & Irritability.	Plusquellec et al., 2010	MH+
11.3 y.o. M 44.9%	Pre and postnatal exposure	Pb levels from umbilical cord and child's blood	GNGT	Impulsive action- P.C. Pb levels_commission error // Impulsive action & attention- N.C. cord blood Pb levels_general task performance // N.C. postnatal Pb levels_P3 ERP component (NoGo)	Boucher et al., 2012a	MH+
11.3 y.o. M 49.5%	Pre and postnatal exposure	Pb levels from umbilical cord and child's blood	The Teacher Report Form; Disruptive Behavior Disorders Rating Scale; DSM-IV	Postnatal Pb levels associated to hyperactive/impulsive ADHD type.	Boucher et al., 2012b	MH+
9.1 y.o. M 52.6%	Postnatal exposure	Pb levels from child's blood	CPT; ADHD-RS (parents and teachers)	P.C. Postnatal Pb levels_Impulsivity/hyperactivity	Hong et al., 2015	MH+
7.7 y.o. M 85.1%	Postnatal exposure	Pb levels from urine	DSM-IV-TR; K-SADS-E; SNAP-IV Scale both parent and teacher forms	P.C. Postnatal Pb levels_Impulsivity & hyperactivity& attention ADHD types	Lee et al., 2018	M+
6-15 y.o. M (N.I.)	Postnatal exposure	Pb levels from child's blood	No-spatial discrimination reversal task	Compulsivity, inflexibility- High exposed > CNT // Learning- High exposed < CNT // Sensory, motor & motivation- Exposed = CNT	Evans et al., 1994	ML+
