# Supplementary material for: A Systematic Review on the Influences of Neurotoxicological Xenobiotic Compounds on Inhibitory Control
Source: Front Behav Neurosci. 2019 Jul 4;13:139. doi: 10.3389/fnbeh.2019.00139 (PMC6620897; doi:10.3389/fnbeh.2019.00139)
Supplement: Supplementary file 3 [file Data_Sheet_3.PDF]

Age, Sex, Strain	Dose & Exposure Time	Exposure Control	Behavioral test/Questionnaires	Behavioral/Pharmacological/Physiological outcomes	Reference	Quality Index
PNW6-16<br>M 0%<br>Mice C57BL/6	4-6 mg/kg/day<br>GD7-9/12-14	Hg from liver & brain	DAT	Compulsivity, perseveration, working memory- Early gestational exposed & Late gestational high exposed > CNT; such effects were blocked with the delays // Motor- Exposed=CNT	Doré et al., 2001	H+
PNW6<br>M 50%<br>Mice C57BL/6	4, 6 & 8 ppm<br>GD2-PND21	MeHg from liver & brain	DAT	Compulsivity, perseveration, working memory- Exposed = CNT // Motor- Exposed females < CNT	Goulet et al., 2003	H?
PNM 9-12<br>M 0%<br>LE	40 or 400 ug/kg/day<br>≈2 weeks prior mating to PND16	N.I.	Multiple fixed ratio; DRL; progressive ratio schedules of reinforcement	Impulsivity- Exposed > CNT at middle FRs; High exposed > rest early stages DRL // High exposed > rest low rate demand progressive ratios	Paletz et al., 2006	H-
PND90<br>M 100%<br>Mice C57BL/6	0.3 or 3 ppm<br>PND21-59	MeHg from brain	DDT	Impulsive choice- Exposed > CNT // Reinforce magnitude sensitivity- Low exposed < CNT	Boomhower and Newland, 2016	H+
PND90<br>M 100%<br>Mice C57BL/6	3 ppm<br>PND21-59	N.I.	Spatial discrimination reversal task; Visual discrimination with extradimensional shift	Compulsivity, inflexibility- Exposed > CNT // Compulsivity, perseveration- Exposed+amphetamine > CNT+amphetamine // Compulsivity, inflexibility with attentional shifting- Exposed > CNT, this effect was blocked with amphetamine.	Boomhower and Newland, 2017	H+
PND24<br>M 50%<br>LE	1,2-4 mg/kg/day from GD6-15	Functional battery	DAT	Compulsivity, perseveration, motor- Exposed = CNT	Goldey et al., 1994	MH?
PNM4-6<br>M 0%<br>LE	0.5-6.4 ppm from 2 prior mating- PND16	MeHg levels from brain	Multiple DRH	Impulsive action- Exposed = CNT (steady state); Sensitivity to DA agonist- High exposed > CNT; Sensitivity to GABA agonist- High exposed < CNT // Learning- Exposed = CNT.	Rasmussen & Newland, 2001	MH-
1.7 & 2.3 y.o.<br>M ≈50%<br>LE	0.5, or 6.4 ppm from 28 or 49 days prior mating-PND16	MeHg from brain	Multiple random intervals transitory schedule of reinforcement tasks	Compulsivity, inflexibility- Exposed > CNT // Learning- Exposed < CNT (2.3 y.o.)	Newland et al., 2004	MH?
PND110<br>M50%<br>LE	0.5 ppm<br>28 days before mating - PND16	N.I.	Spatial reversal learning task (cued / non-cued); DAT	Compulsivity, perseveration, working memory- Exposed > CNT (non-cued & delayed)	Widholm et al., 2004	MH+
PNM26<br>M 50%<br>Mice B6C3F1/HSD _ CBA/<br>J HSD	1-3 ppm perinatal (until PND13)-lifetime	MeHg levels from brain	DAT	Compulsivity, perseveration, working memory- Exposed > CNT & Low exposed (longer delays)	Weiss et al., 2005	MH?
PNM1<br>M 50%<br>Mice ARE-hPAP/<br>C57BL/6/Bkl	0.5 mg/kg/day from GD7-PND7	MeHg levels from brain	Place learning and reversal.	Compulsivity, inflexibility- Exposed > CNT // Motor- Exposed = CNT	Onishchenko el al., 2007	MH-

PNM13-20<br>M 0%<br>LE	40 or 400 ug/kg/day<br>≈2.5 weeks prior mating to PND16	N.I.	Fixed ratio/interval schedule of reinforcement paradigm; Alternating /Clocked fixed schedule of reinforcement; Peak interval procedure (non-reinforced responses)	Impulsive action- High exposed > rest (FI/CFI); decreased response rate High exposed by age in CFI // Compulsivity, perseveration- High exposed > rest (non-reinforced stage)	Reed & Newland, 2007	MH?
PNM6<br>PNM13-21<br>M 50.9%<br>LE	40 or 400 ug/kg/day<br>≈2.5 weeks prior mating to PND16	N.I.	Multiple fixed ratio schedule of reinforcement and progressive ratio	Impulsivity - High exposed > rest at lower ratio; High exposed + Se diet > rest at lower ratio/higher increased reinforce magnitude; Low exposed + low Se < rest at larger ratio; High exposed + high Se diet > rest	Reed et al., 2008	MH?
PNM10-12<br>M 0%<br>LE	40 or 400 ug/kg/day<br>≈2.5 weeks prior mating to PND16	N.I.	Fixed ratio/interval schedule of reinforcement; Alternating/ Clocked fixed interval schedule of reinforcement	Impulsive action following low Se diet- Low exposed < High exposed (F) Cocaine exposure; MeHg increased sensitivity to DA challenge	Reed & Newland, 2009	MH?
PND104<br>M 50%<br>LE	1.5-4.5 ppm<br>28 Days before breeding-PND21	Functional battery	DRL	Impulsive action- Exposed = CNT; Sensitivity to DA challenge= Exposed + amphetamine < CNT + Amphetamine; MeHg blocked PCB effects on impulsive action.	Sable et al., 2009	MH+
7-9 y.o<br>M (NI).<br>MF	50, 70 - 90 ug/kg/day throughout pregnancy	MeHg from blood	DAT	Compulsivity, perseveration, working memory- Exposed < CNT	Gilbert et al., 1993	M-
5-6 y.o.<br>M 100%<br>SM	0.7-0.9 ppm/day<br>GW11 or 14.5 - final gestation	MeHg from brain & blood	Multiple random interval transitory schedule of reinforcement	Compulsivity, inflexibility- Exposed > CNT // Learning- Exposed < CNT	Newland et al., 1994	M-
PND90-2.5 y.o.<br>M 0%<br>LE	0.5-6.4 ppm from 28/49 days prior mating - PND16	MeHg levels from brain	Multiple DRH	Impulsive action - High exposed > CNT decline defined by age; BL learning- Exposed = CNT	Newland & Rasmussen, 2000	M+
PNM 11-13<br>M 0%<br>LE	400 ug/kg/day<br>≈2.5 weeks prior mating to PND16	N.I.	Spatial discrimination reversal task	Compulsivity, inflexibility- High exposed > CNT // Learning- Exposed = CNT	Reed et al., 2006	M?
PNM 15-18 and 24-27<br>M 0%<br>LE	40 or 400 ug/kg/day<br>≈2 weeks prior mating to PND16	MeHg from brain (ppm)	Spatial & visual discrimination reversal tasks	Spatial. Compulsivity, inflexibility- Exposed > CNT at early reversal; no observed in elder // Learning- High exposed < rest; Visual. Compulsivity, inflexibility- High exposed > rest	Paletz et al., 2007	M-
Adulthood<br>M 0%<br>LE	40- 400ug/kg/day<br>18 weeks prior mating-PND16	MeHg from brain	PCNT-H 20:0.75-high-rate schedule; PCNT-L 20:0.75-low-rate schedule	Impulsivity action- High exposed > CNT. Compulsivity, inflexibility & perseveration- High exposed > CNT; No effects of Se diet	Newland et al., 2013	M-
