# Supplementary material for: A Systematic Review on the Influences of Neurotoxicological Xenobiotic Compounds on Inhibitory Control
Source: Front Behav Neurosci. 2019 Jul 4;13:139. doi: 10.3389/fnbeh.2019.00139 (PMC6620897; doi:10.3389/fnbeh.2019.00139)
Supplement: Supplementary file 4 [file Data_Sheet_4.PDF]

Age & Sex	Dose & Exposure Time	Exposure Control	Behavioral test/Questionnaires	Behavioral/Pharmacological/Physiological outcomes	Reference	Quality Index
18-74 y.o. M 52.7%	Postnatal exposure	Hg/MeHg levels from both hair and blood; Fish intake	BSI	P.C. MeHg levels_obsessive/compulsive symptoms in women	Phillibert et al., 2008	H+
11.2 y.o. M 67%	Pre and post-natal exposure	MeHg levels from umbilical cord and child's blood	Visuospatial attention-shift paradigm;	Impulsive action- Exposed = CNT	Ethier et al., 2015	H+
22-24 y.o. M ≈47.5%	Pre & postnatal exposure	MeHg from participant and participant's mother hair	Intra/extra dimensional shift; Rapid visual information processing; Barkley adult ADHD rating scale; Test of variables of attention visual/auditory	22 y.o. P.C. Postnatal exposure_ Compulsive rates (total errors IED) // 24y.o. N.C. Gestational exposure_ attentional variables (auditory) // Impulsivity- no related to MeHg exposure	van Wijngaardeen et al., 2017	H+
>17 y.o. M40.3%	Postnatal exposure	MeHg levels from hair Exposure and occupation time	Concentrated Attention Test of the Toulouse Pierron Factorial Battery	P.C. MeHg levels_impulsive action. No mood effects.	Yokoo et al., 2003	MH+
4.5 y.o. M 45%	Gestational exposure	Hg levels from mothers' hair	Michigan Catch-the-Cat Test (CPT)	No relation between MeHg levels_Impulsive action // N.C. Corpus Callosum volume (Splenium)_Impulsivity rates.	Stewart et al., 2003	MH+
8-9.5 y.o. M (N.I.)	Gestational exposure	Hg from mothers' hair	NES2 (CPT)	No relation between MeHg levels_Impulsive action	Stewart et al., 2005	MH+
9.5 y.o. M (N.I.)	Gestational exposure	Hg levels from mother's hair	DRL	N.C. Prenatal & postnatal levels_Money earned // P.C. early/late gestational exposed _Impulsive action, even after PCB exposure co-factor controlled	Stewart et al., 2006	MH+
9.9 y.o. M 51%	Postnatal exposure	Hg levels from blood	KITAP (alertness, GNGT, distract- ability and flexibility subtests); ICD-10; DSM-IV; FBB-ADHS	No relation between Postnatal Hg levels_Impulsivity or compulsivity	Nicolescu et al., 2010	MH+
5.4 y.o. M 44.6%	Pre and post-natal exposure	Pb levels from umbilical cord and child's blood	Infant Behavior Rating Scale	No relation between MeHg levels and impulsivity rates	Plusquellec et al., 2010	MH+
11.3 y.o. M 44.9%	Pre and post-natal exposure	MeHg levels from umbilical cord and child's blood	GNGT	Impulsive action- No effects following covariates control	Boucher et al., 2012a	MH+
11.3 y.o. M 49.5%	Pre and Post-natal exposure	MeHg levels from umbilical cord and Child's blood	The Teacher Report Form; Disruptive Behavior Disorders Rating Scale; DSM-IV	Impulsive ADHD type- gestational exposed=CNT // Inattentive ADHD type- Gestational exposed > CNT	Boucher et al., 2012b	MH+

14 y.o.<br>M ≈50%	Pre & post-natal exposure	PCB levels from umbilical cord tissue	NES2-CPT	P.C. Gestational exposure_RT // No effects on impulsive action	Debes et al., 2006	M+
7.7 y.o.<br>M 85.1%	Postnatal exposure	Hg levels from urine samples	DSM-IV-TR; K-SADS-E; SNAP-IV both parent and teacher forms	P.C. Postnatal Hg levels_ Impulsive & hyperactive ADHD type (parents referred)	Lee et al., 2018	M+
