# Supplementary material for: A Systematic Review on the Influences of Neurotoxicological Xenobiotic Compounds on Inhibitory Control
Source: Front Behav Neurosci. 2019 Jul 4;13:139. doi: 10.3389/fnbeh.2019.00139 (PMC6620897; doi:10.3389/fnbeh.2019.00139)
Supplement: Supplementary file 5 [file Data_Sheet_5.PDF]

Age & Sex & Strain	Dose & Exposure Time	Exposure Control	Behavioral test/Questionnaire	Behavioral/Pharmacological/Physiological outcomes	Reference	Quality Index
PND380 M 100% WR	5 or 30mg/kg/day 60 days before mating -onwards.	PCB levels from brain and adipose tissue	Continuous fixed interval schedule of reinforcement	Impulsive action- High exposed > rest // Motor- High exposed > rest	Lilienthal et al., 1990	H+
PND 165 M 50% SD	PCB28_ 8-32, PCB118_4-16 & PCB153_16-64mg/kg/day from GD10-16	Reproductive & developmental battery	DAT	Learning, attention- High exposed females (3 congeners) < CNT females // No analysis of perseveration	Schantz et al., 1995	H-
PNW5 M 100% DA/OLA/HSD	5 mg/kg/day (PCB153) or 2ug/kg (PCB126). PND3-13 (Every two days)	PCB levels from brain, liver & stomach; Functional battery.	Multiple fixed interval/extinction schedule of reinforcement	Impulsive action & compulsivity, perseveration - Exposed> CNT	Holene et al., 1998	H+
PND60 M 50% LE	0.25-1 ug/kg/day from 35 days prior mating-PND21	PCB levels from fat tissue, blood & brain; Functional battery	Multiple fixed interval/ratio schedule of reinforcement; DRL	Impulsive action- Exposed = CNT	Rice & Hayward 1998	H?
PND60 M 50% LE	0.25-1 ug/kg/day from 35 days prior mating-PND21	PCB levels from fat tissue, blood & brain; Functional battery	DAT	Compulsivity, perseveration, learning- Exposed = CNT	Rice 1999	H+
PND23 M 50% LE	Aroclor 1254_8, Aroclor1016_10 mg/kg from GD6-parturation; GD6-PND21	Brain & body weight control	DAT	Learning, attention- Exposed = CNT // Norepinephrine brain levels- Exposed < CNT (Aroclor 1254)	Zahalka et al., 2001	H?
Adulthood M 57.8% SD	31 ppm from PND35--64	PCB levels from fat tissue	Multiple fixed interval/extinction schedule of reinforcement	Impulsive action- Exposed > CNT in males (larger rates for sediment contaminated)	Lombardo et al., 2015	H+
Adulthood M 50% LE	3, 6 mg/kg/day PND27-50	N.C.	Set-Shifting task (Visual discrimination & Position discrimination & reversal learning); DRL	Impulsive action- Low exposed > rest (first DRL session) // Compulsivity, inflexibility- High exposed (males) < CNT	Monaikul et al., 2017	H+
PNM 14 M22% RM	Variable dose range 0.25-2.5 ppm in variable time schedule both maternal & gestational & postnatal	PCB levels from Maternal fat, milk and offspring fat	Discrimination spatial/non-spatial reversal learning with irrelevant cues (shape and color).	Exp1. Aroclor 1248. Compulsivity, inflexibility- Exposed = CNT // Exp2. Aroclor 1016. Compulsivity, inflexibility- Exposed = CNT (Spatial); Exposed < CNT (shape) // Learning- High exposed < CNT (spatial)	Schantz et al., 1989	MH-
PND 135 M 50% SD	PCB77_2-8, PCB126_0.25-1 mg/kg/day from GD10-16	Reproductive & developmental battery;	DAT	Learning, attention, compulsivity- Exposed = CNT // No analysis of perseveration	Schantz et al., 1996	MH+
PND400 M 50% LE	0.25-1 ug/kg/day from 35 days prior mating-PND21	PCB levels from fat tissue, blood & brain; Functional battery	Concurrent R1-R1, continuous reinforcement schedule; Progressive ratio	Impulsive action- Exposed = CNT // Learning- High exposed <CNT	Rice & Hayward, 1999b	MH+
Adults M 100% SD	Diet 0.5ug/g Aroclor 1248. Fish 1.15g/27.40g	PCB levels from fat tissue	Multiple continuous fixed interval/extinction schedule of reinforcement	Impulsive action- Exposed (both) > CNT (late segments, burst) // Compulsivity, perseveration- Exposed = CNT	Berger et al., 2001	MH+

≈ PND235<br>M 50%<br>LE	1, 3, 6 mg/kg/day<br>28 Days before breeding-PND21	N.C.	DRH; DRL/extinction	Impulsive action- exposed (middle & high) > CNT (DRH) // Compulsivity, perseveration- High exposed > CNT	Sable et al., 2006	MH+
PND104<br>M 50%<br>LE	1, 3 mg/kg/day<br>28 Days before breeding-PND21	Functional battery	DRL	Impulsive action- Exposed > CNT (eminently in males); Hyposensitivity to DA agonist challenge	Sable et al., 2009	MH+
PND25<br>M 100%<br>WR Kyoto	PCB 52, 153 & 180_10mg/kg at PND8, 14 & 20	N.I.	Multiple variable interval schedule of reinforcement	General activation-PCB153&180 Exposed < CNT&PCB52 // Impulsive action- PCB52 Exposed > rest // Learning- Exposed= CNT	Johansen et al., 2011	MH+
PND100<br>M 50%<br>LE	3, 6 mg/kg/day<br>28 days prior mating-PND21	Functional battery	DRL	Impulsive action- Low exposed > CNT females	Meyer et al., 2015	MH+
4 y.o.<br>M 100%<br>MF	7.5ug/kg/day<br>From birth to PNW20.	PCB levels from both pups' blood and adipose tissue	Multiple fixed interval/ratio schedule of reinforcement	Impulsive action- Exposed > CNT (FI)	Rice 1997b	M+
4.5-5 y.o.<br>M 100%<br>MF	7.5ug/kg/day<br>From birth to PNW20.	PCB levels from both pups' blood and adipose tissue	DRL; Discrimination spatial reversal with irrelevant cues	Impulsive action & hyperactivity- Exposed > CNT // Compulsivity, inflexibility- Exposed=CNT (albeit some PCB animals did perform worse).	Rice, 1998	M+
8-24 m.o.<br>M	2.5ppm Aroclor 1248 to mothers until weaning	PCB levels from adipose tissue	Discrimination spatial and non-spatial (Color & Form) reversal test; Probability learning (shifts); Object alternation learning	P.C. PCB levels_Hyperactivity & inflexibility. // Compulsivity, inflexibility- Exposed > CNT (spatial & color conditions), perseveration Exposed > CNT (shift)	Bowman et al., 1978	ML-
3 y.o.<br>M 100%<br>MF	7.5ug/kg/day<br>From birth to PNW20.	PCB levels from both pups' blood and adipose tissue	Discrimination reversal (non-spatial); DAT	Compulsivity, inflexibility & learning - Exposed > CNT	Rice & Hayward, 1997	ML+
5 y.o.<br>M 100%<br>MF	7.5ug/kg/day<br>From birth to PNW20.	PCB levels from both pups' blood and adipose tissue	Concurrent R1-R1, continuous reinforcement schedule; Progressive ratio (FR)	Impulsive action- Exposed > CNT (only in FR progressive schedule)	Rice & Hayward, 1999a	ML+
≈ PND235<br>M 50%<br>LE	6mg/kg/day<br>GD6-PND21	N.C.	Spatial discrimination reversal learning	Compulsivity, inflexibility- Exposed males (early reversal) > CNT // Attentional, associative- Exposed females (late reversal) > CNT	Widholm et al., 2001	ML?
PND25<br>M 100%<br>SHR/NCrl	PCB 153_ 1, 3-6 mg/kg at PND8, 14 & 20	N.I.	Multiple variable interval schedule of reinforcement	General activation- High exposed > CNT // Impulsive action- Middle exposed > Low exposed (females' hypertensive rats)	Johansen et al., 2014	ML?
