# Supplementary material for: A Systematic Review on the Influences of Neurotoxicological Xenobiotic Compounds on Inhibitory Control
Source: Front Behav Neurosci. 2019 Jul 4;13:139. doi: 10.3389/fnbeh.2019.00139 (PMC6620897; doi:10.3389/fnbeh.2019.00139)
Supplement: Supplementary file 6 [file Data_Sheet_6.PDF]

Age & Sex	Dose & Exposure Time	Exposure Control	Behavioral test/Questionnaire	Behavioral/Pharmacological/Physiological outcomes	Reference	Quality Index
11.2 y.o. M 66.7%	Pre and Post-natal exposure	PCB levels from umbilical cord blood and child's sample	Visuospatial attention-shift paradigm	Impulsivity: No effects // Attention- N.C. Gestational PCB levels_Attentional performance & Speed processing	Either et al., 2015	H+
4.5 y.o. M 45%	Gestational exposure	PCB levels from umbilical cord	Michigan Catch-the-Cat Test	P.C. PCB levels_Impulsive action // N.C. Corpus Callosum volume (Splenium)_Impulsivity rates.	Stewart et al., 2003	MH+
4 & 11y.o. M 52.7%	Pre and post-natal exposure	PCB levels from umbilical cord, breast milk and mother's serum;	CPT; WCST	P.C. PCB levels_Impulsive action & Compulsive perseveration (11 y.o. group) for < 6 weeks of breast feeding // Impulsivity- General larger levels in relation with Gestational exposure.	Jacobson & Jacobson, 2003	MH+
8 & 9.5 y.o. M (N.I.)	Gestational exposure	PCBs, DDE and HCB Umbilical cord blood	NES2-CPT; (E)-CPT	P.C. PCB levels_Impulsive action (8 y.o.); also, at 9.5 y.o. at high-demanded responses.	Stewart et al., 2005	MH+
9.5 y.o M (N.I.).	Gestational exposure	PCBs, DDE and HCB Umbilical cord blood	DRL	N.C. PCB total levels_money earned	Stewart et al., 2006	MH+
9 y.o. M ≈ 52%	Pre & postnatal exposure	PCB levels from umbilical cord blood, milk and mothers' blood	SRTT	Impulsive action, attention- High exposed > Low exposed	Vreugdenhil et al., 2004	MH+
5.4 y.o. M 44.6%	Pre & post-natal exposure	PCB levels from umbilical cord blood and child's blood	Infant Behavior Rating Scale	Impulsivity- no relation with PCB exposure // P.C. unhappiness & anxiety_PCB levels	Plusquellec et al., 2010	MH+
8 y.o. M 51.2%	Gestational exposure	PCB & DDE levels from umbilical cord blood	Conner's Rating Scale for teachers; DSM-IV	P.C. PCB & p,p'-DDE gestational levels_risk ADHD-like outcomes	Sagiv et al., 2010	MH+
11.3 y.o. M 44.9%	Pre and post-natal exposure	PCB levels from umbilical cord and Child's blood	GNGT	Impulsive action- No effects in relation to PCB exposure //Attention- P.C. Postnatal PCB levels_RT // N.C. Pe/Pc amplitude_PCB concurrent exposure (Anterior cingulate cortex)	Boucher et al., 2012a	MH+
11.3 y.o. M 49.5%	Pre and post-natal exposure	PCB levels from umbilical cord and Child's blood	The Teacher Report Form; Disruptive Behavior Disorders Rating Scale; DSM-IV	Impulsivity & hyperactivity- No relation with exposure	Boucher et al., 2012b	MH+
4 & 11 y.o. M 49.4%	Pre and Post-natal exposure	PCBs and DDE levels from umbilical cord blood & child's blood	CPT-II	N.C. PCB & DDE levels (4 y.o.) Speed processing // Protective effects of breastfeeding	Forns et al., 2012	MH+
8 y.o. M ≈50%	Gestational exposure	PCBs and DDE levels from umbilical cord blood	NES2-CPT	N.C. PCB/DDE levels_attentional performance & speed processing (eminently in males) // No effects on inhibition	Sagiv et al., 2012	MH+
8 y.o. M ≈ 50%	Pre & postnatal exposure	PCB levels from umbilical cord blood, child's blood & fatty tissue	Conners' Rating Scale for teachers; DSM-IV	P.C. gestational exposure level_hyperactivity & impulsivity; also, in postnatal but reduced by time	Verner et al., 2015	MH+
18.1 y.o. M 36.4%	Postnatal exposure	PCB levels	Conners' CPT-II	Attention- Exposed men < CNT // Impulsivity- Exposed = CNT	Behforooz et al., 2017	MH+

44 y.o.<br>M 84.8%	Postnatal exposure	PCB levels from blood	TMT	No effects PCB burden rate_inflexibility	Fimm et al., 2017	M+
