# Supplementary material for: A Systematic Review on the Influences of Neurotoxicological Xenobiotic Compounds on Inhibitory Control
Source: Front Behav Neurosci. 2019 Jul 4;13:139. doi: 10.3389/fnbeh.2019.00139 (PMC6620897; doi:10.3389/fnbeh.2019.00139)
Supplement: Supplementary file 7 [file Data_Sheet_7.PDF]

Age & Sex & Strain	Dose & Exposure Time	Exposure Control	Behavioral test/Questionnaires	Behavioral/Pharmacological/Physiological outcomes	Reference	Quality Index
Adulthood M 100% LE	DFP 1mg/kg, Disulfoton 2mg/kg/day for 14 days	AChE activity from different brain areas	Spontaneous alternation test	Compulsivity, perseveration, working memory- Exposed > CNT // Muscarinic binding at cortex, hippocampus and striatum- Exposed < CNT	McDonald et al., 1988	H+
PND22-24; 61-91 M 50% SD	CPF 0.3, 1 - 5 mg/kg GD6-PND10	CPF levels in blood and milk; TCP levels in blood; ChE activity erythrocytes, heart and brain; Functional battery	DAT	Compulsivity, perseveration- Exposed = CNT	Maurissen et al., 2000	H?
PNM4 M100% WR	CPF 166-250mg/kg Px 0.4-1mg/kg PNW16 & 38	ChE activity; Functional battery	DAT	1 exposure- Compulsivity, perseveration & learning - Exposed = CNT // 2 exposure- Task performance- Exposed < CNT at early days // Compulsivity, perseveration- no specification // Motor- Heavily affected	Sánchez-Santed et al., 2004	H+
PND35 M 50% SD	PTN 0.1-0.2 mg/kg/day from PND1-4	Functional battery	Alternation task	Learning, attention, Compulsivity, perseveration- Exposed = CNT Motor- Exposed = CNT	Timofeeva et al., 2008	H+
PNM5,17 M 100% WR	CPF 250 mg/kg acute at PNM3	AChE activity whole brain & functional battery	DDT; SIP	Impulsive choice- Exposed > CNT (Short-term) // Exposed HD > rest (long-term) // Compulsivity, adjunctive behavior- Exposed > CNT	Cardona et al., 2011	MH+
Adulthood M 100% WR	CPF 5 mg/kg/day for 31 weeks	AChE activity from half hemisphere; Functional battery	DDT	Impulsive choice- Exposed > CNT // AChE-R Expression > CNT	Lopez-Granero et al., 2013	MH+
PNM6-14 M 100% WR	CPF 250 mg/kg DFP 1.5 mg/kg PTN 15 mg/kg PNM3	AChE-S activity; Functional battery	DDT	Impulsive choice- Exposed = CNT // Short-term AChE-S expression- Exposed < CNT // Long-term AChE-R expression- Exposed (CPF/DFP) < CNT	Lopez-Granero et al., 2014	MH-
PNW≈9 M 100% LH	CPF/ DZN 1 mg/kg for 5 days	AChE activity in different brain structures and blood	MBT	Compulsivity- Exposed < CNT // DA levels frontal & Striatum- Exposed < CNT // 5HT-T levels Frontal and Hippocampus- Exposed < CNT	Savy et al., 2015	MH+
Adulthood F 100% APOE2, 3 and 4 mice	CPF 3.75 mg/kg/day dietary for 4 weeks after BL	ChE activity both plasma and FC	5-CSRTT	CPF exposure. Impulsive action- Exposure < BL // Compulsivity, perseveration- CPF blocked APOE4 increased perseveration at BL // GABA agonist (Alprazolam) did not affect impulsive/compulsive traits	Peris-Sampedro et al., 2016	MH-
> 3 m.o. M100% WR	DFP 0.5 mg/kg/day PNM3-4 30 consecutive days exposure	Functional battery; plasma and whole brain ChE activity	5-CSRTT	Impulsive action- Exposed < CNT (exposure period) larger ITI; Exposed > CNT (Washout period) larger ITI // Compulsivity, inflexibility- Exposed > CNT (Exposure/Wash-out), stronger at lower SD (Exposure)	Terry et al., 2014	MH+
Adulthood M 100% LH	DZN 1 and 2mg/kg/day, 5	A/BuChE activity in different brain structures and blood	MBT	Compulsivity- Exposed < CNT	Savy et al., 2018	MH+

	days/week, 12 weeks					
Adulthood<br>M 100%<br>LE	DFP 1 + 0.5 mg/kg	AChE activity from brain cortex	Discrimination reversal task	Compulsivity, inflexibility- DFP + Scopolamine > Saline + Scopolamine //	Raffaele et al., 1990	M?
PNM9<br>M 100%<br>WR	CPF 250 mg/kg acute at PNM3	AChE activity whole brain & functional battery	SIP; DDT	Impulsive choice- Exposed > CNT; Exposed HD > CNT HD // Compulsivity, adjunctive behavior- Exposed HD > CNT HD; GABA agonist increased compulsivity only in CPF HD.	Cardona et al., 2006	M+
PNM2<br>M 100%<br>WR	CPF 18 mg/kg. sub chronic 14 or 30 consecutive days PNM2	ChE activity different brain areas	5-CSRTT	14 d.e. Impulsive action- Exposed > CNT (always) // Compulsivity, perseveration- Exposed < CNT (exposure period)<br>30 d.e. Impulsive action- Exposed > CNT (always) // Compulsivity, perseveration- Exposed < CNT (exposure period); Exposed > CNT (Wash out)	Middlemore-Risher et al., 2010	M+
PND45<br>M ≈ 50%<br>ICR Mice	CPF 1-5 mg/kg GD13-17	N.I.	DAT	Compulsivity, perseveration- High exposed -CNT at longer delays (males) and early (females) // CA1 & DG & Prl & IL cells number- High exposed < CNT // Muscarinic binding at cortex & hippocampus- Exposed < CNT	Chen et al., 2012	M+
PND200<br>M 100%<br>WR	CPF_ 10-18, DFP_0.25-0.75 mg/kg/day from PND30-60	ChE activity from brain & blood	Water maze with reversal	Compulsivity, inflexibility- High exposed > CNT (DFP) // Learning- High exposed < CNT (CPF), Exposed < CNT (DFP)	Terry et al., 2012	M+
Adulthood<br>M 100%<br>LH	CPF 250 mg/kg acute	AChE activity; Function battery	5-CSRTT	Impulsive action- CPF = CNT // Compulsivity, perseveration- Exposed > CNT (BL); Amphetamine reduced compulsivity in CPF, no in CNT // DOPAC/DA & DOPAC+HVA/DA activity- Exposed > CNT (Hippocampus) // GABA & Glutamate activity- Exposed < CNT (Striatum)	Montes de Oca et al., 2013	M?
