# Supplementary material for: A Systematic Review on the Influences of Neurotoxicological Xenobiotic Compounds on Inhibitory Control
Source: Front Behav Neurosci. 2019 Jul 4;13:139. doi: 10.3389/fnbeh.2019.00139 (PMC6620897; doi:10.3389/fnbeh.2019.00139)
Supplement: Supplementary file 8 [file Data_Sheet_8.PDF]

Age & Sex	Dose & Exposure Time	Exposure Control	Behavioral test/Questionnaires	Behavioral/Pharmacological/Physiological outcomes	Reference	Quality Index
3.5 & 5 y.o. M 46.8%	Pre & postnatal exposure	OP metabolites from urine of the mother (pregnancy) and children	Child behavior checklist; NEPSY-II, Conner's's' Kiddie CPT	Impulsivity- No effects // P.C. Exposure_ attentional alterations	Marks et al., 2010	H+
29 y.o. M 100%	N.I.	Number of poisonings and ChEs activity from blood	BSI Test battery of neurobehavioral performance. Depression dimension.	Impulsivity, suicide ideation- Exposed > CNT; Non-observed in Carbamate exposed // Mood alterations amongst exposed	Wesseling et al., 2010	H+
6.1y.o. M 53.4%	Postnatal exposure	Household Methyl-Paranthion levels and P*ara*Nitropheo levels in urine	Pediatric Environmental Neurobehavioral Battery; Personality Inventory for Children	Impulsivity, altered self-control (informant-based)- Exposed > CNT	Ruckart et al., 2004	MH+
10.3 y.o. M 61.5%	Postnatal exposure	Number of OP acute exposure symptoms	NEPSY (Statue and Knock Tap subtests)	Impulsive action- Exposed> CNT (statue)	Kofman et al., 2006	MH+
7-11 y.o. M 48%	Gestational exposure	TCPY levels from mother's urine	Behavior Assessment System for Children; Conners' Parental Rating Scale revised & CPT	Impulsivity- No significant effects	Fonteberry et al., 2014	MH+
15.3 y.o. M	Postnatal exposure	BChE activity from blood; TCPy levels from urine; Functional examination; History of exposure	TMT	Compulsivity, flexibility- Exposed = CNT	Ismail et al., 2017	MH+
26.4 y.o. M 50%	Postnatal exposure	History of exposure; AChE activity	TMT; Verbal fluency	Compulsivity, flexibility- Exposed > CNT; 3 months later, only in verbal fluency // Brain blood flow alteration in exposed participants, right hemisphere and occipital areas.	Mittal et al., 2011	M+
≈ 6.6 y.o. M ≈ 51%	Postnatal exposure	AChE activity; Time (days) after exposure period and	NEPSY-II test	Impulsive action- proximal days exposure > later ones	Suarez-Lopez et al., 2017	M+
<34->50 y.o M 55.6%	Postnatal exposure	Self-reported OP storage at home	12-item General Health Questionnaire	Impulsivity, suicide ideation- OP storers > No OP storers.	Zhang et al., 2009	ML+
54.7 y.o. M 80.3%	Postnatal exposure	Time working with OPs and years from last dip	Trails B; CALCAP Choice; Stroop test; Verbal fluency	Impulsivity, altered strategy making- Exposed > CNT // Compulsivity. inflexibility- Exposed > CNT // Mood disorders across exposed group	Mackenzie Ross et al., 2010	ML+
<60>70 y.o. M 58.7%	N.I.	Cultivated land area, annual exposure time, neurotoxicity symptoms and farmer's syndrome	Center for Epidemiologic Studies Depression Scale; Swedish Q16	Impulsivity, suicide ideation- Exposed women > Exposed men; P.C. History of neurotoxicity and farmer's syndrome mood alteration/suicidal ideation	Joo & Roh, 2016	ML+
15.7 y.o. M 100%	Postnatal exposure	History of exposure; Metabolites levels from urine	TMT; Reversal learning	Compulsivity, perseveration- High exposed > Low exposed, albeit no effect on reversal learning	Rohlman et al., 2016	ML+
7 y.o. M 44%	Postnatal exposure	OP metabolites (Dialkylphosphates) in urine.	WCST	P.C. OP metabolites concentration_Compulsive perseveration	Sanchez Lizardi et al., 2008	L+
