# Supplementary material for: A Systematic Review on the Influences of Neurotoxicological Xenobiotic Compounds on Inhibitory Control
Source: Front Behav Neurosci. 2019 Jul 4;13:139. doi: 10.3389/fnbeh.2019.00139 (PMC6620897; doi:10.3389/fnbeh.2019.00139)
Supplement: Supplementary file 9 [file Data_Sheet_9.pdf]

Table 1a. Summary of the main published preclinical studies on the link between lead exposure and impulsive/compulsive traits. The studies are organized based on quality index score from the highest (top) to the lowest rate (bottom). The studies from the same quality category are organized based on publication date. M= males, RM= Rhesus Monkeys, y.o.= years old, m.o.= months old, M.F.= Macaca Fascicularis, L.E.= Long Evans, SD= Sprague Dawley, PND/W/M= postnatal day, week, month, N.I.= non-indicated, >/<= more/less than/equal to, CNT= Control group, DRL= Differential reinforcement to low rates, DAT= Delayed alternation task, MBT= Marble burying test, H/MH/M/ML= High, medium-high, medium and medium-low quality, +/-/?= acceptable, low and not explicit final sample size.

Table 1b. Summary of the main published clinical studies on the link between lead exposure and impulsive/compulsive traits. The studies are organized based on quality index score from the highest (top) to the lowest rate (bottom). The studies from the same quality category are organized based on publication date. M= males, y.o.= years old, m.o.= months old, N.I.= non-indicated, P/N.C.= positive/negative correlation, >/<= more/less than/equal to, CNT= Control group, DRL= Differential reinforcement to low rates, WCST= Wisconsin card sorting test, CPT= Continuous performance test, TMT= Trail making test, GNGT= Go no Go task, K-SADS-E= Kiddie Schedule for Affective Disorders and Schizophrenia, SNAP-IV= Swanson, Nolan and Pelham scale, H/MH/M/ML= High, medium-high, medium and medium-low quality, +/-/?= acceptable, low and not explicit final sample size.

Table 2a. Summary of the main published preclinical studies on the link between methylmercury exposure and impulsive/compulsive traits. The studies are organized based on quality index score from the highest (top) to the lowest rate (bottom). The studies from the same quality category are organized based on publication date. M= males, y.o.= years old, MF= Macaca Fascicularis, LE= Long Evans, PND/W/M= postnatal day, week, month, N.I.= non-indicated, >/<= more/less than, CNT= Control group, DRL/H= Differential reinforcement to low/high rates, DAT= Delayed alternation task, H/MH/M/ML= High, medium-high, medium and medium-low quality, +/-/?= acceptable, low and not explicit final sample size.

Table 2b. Summary of the main published clinical studies on the link between methylmercury exposure and impulsive/compulsive traits. The studies are organized based on quality index score from the highest (top) to the lowest rate (bottom). The studies from the same quality category are organized based on publication date. M= males, y.o.= years old, m.o.= months old, N.I.= non-indicated, P/N.C.= positive/negative correlation, >/<= more/less than/equal to, CNT= Control group, DRL/H= Differential reinforcement to low/high rates, CPT= Continuous performance test, GNGT= Go no Go task, BSI= Brief symptoms inventory, FBB-ADHS= Fremdbeurteilungsbogen für Aufmerksamkeitsdefizit- / Hyperaktivitätsstörungen , K-SADS-E= Kiddie Schedule for Affective Disorders and Schizophrenia , H/MH/M/ML= High, medium-high, medium and medium-low quality, +/-/?= acceptable, low and not explicit final sample size.

Table 3a. Summary of the main published preclinical studies on the link between PCBs exposure and impulsive/compulsive traits. The studies are organized based on quality index score from the highest (top) to the lowest rate (bottom). The studies from the same quality category are organized based on publication date. M= males, RM= Rhesus Monkeys, y.o.= years old, m.o.= months old, MF= Macaca Fascicularis, LE= Long Evans, SD= Sprague Dawley, PND/W/M= postnatal day, week, month, N.I.= non-indicated, N.C.= non-controlled, P/N.C.= positive/negative correlation, >/<= more/less than, CNT= Control group, DRL/H= Differential reinforcement to low/high rates, DAT= Delayed alternation task, H/MH/M/ML= High, medium-high, medium and medium-low quality, +/-/?= acceptable, low and not explicit final sample size.

Table 3b. Summary of the main published clinical studies on the link between PCBs exposure and impulsive/compulsive traits. The studies are organized based on quality index score from the highest (top) to the lowest rate (bottom). The studies from the same quality category are organized based on publication date. M= males, y.o.= years old, N.I.= non-indicated, P/N.C.= positive/negative correlation, >/<= more/less than/equal to, CNT= Control group, DRL/H= Differential reinforcement to low/high rates, WCST= Wisconsin card sorting test, CPT= Continuous performance test, TMT= Trail making test, GNGT= Go no Go task, SRTT= Stop reaction time tasks, H/MH/M/ML= High, medium-high, medium and medium-low quality, +/-/?= acceptable, low and not explicit final sample size.

Table 4a. Summary of the main published preclinical studies on the link between OP exposure and impulsive/compulsive traits. The studies are organized based on quality index score from the highest (top) to the lowest rate (bottom). The studies from the same quality category are organized based on publication date. M= males, LE= Long Evans, SD= Sprague Dawley, PND/W/M= postnatal day, week, month, N.I.= non-indicated, P/N.C.= positive/negative correlation>/<= more/less than/equal to, CNT= Control group, DAT= Delayed alternation task, MBT= Marble burying test, SIP= Schedule-induced polydipsia, 5-CSRTT= 5 choice serial reaction time task, H/MH/M/ML/L= High, medium-high, medium, medium-low and low quality, +/-/?= acceptable, low and not explicit final sample size.

Table 4b. Summary of the main published clinical studies on the link between OP exposure and impulsive/compulsive traits. The studies are organized based on quality index score from the highest (top) to the lowest rate (bottom). The studies from the same quality category are organized based on publication date. M= males, y.o.= years old, N.I.= non-indicated, P/N.C.= positive/negative correlation>/<= more/less than/equal to, CNT= Control group, WCST= Wisconsin card sorting test, CPT= Continuous performance test, TMT= Trail making test, H/MH/M/ML/L= High, medium-high, medium, medium-low and low quality, +/-/?= acceptable, low and not explicit final sample size.
